# Integrating Spatial Modelling and Space–Time Pattern Mining Analytics for Vector Disease-Related Health Perspectives: A Case of Dengue Fever in Pakistan

**DOI:** 10.3390/ijerph182212018

**Published:** 2021-11-16

**Authors:** Syed Ali Asad Naqvi, Muhammad Sajjad, Liaqat Ali Waseem, Shoaib Khalid, Saima Shaikh, Syed Jamil Hasan Kazmi

**Affiliations:** 1 Department of Geography, Government College University Faisalabad, Faisalabad 38000, Pakistan; drliaqataliwaseem@gcuf.edu.pk (L.A.W.); shoaibkhalid@gcuf.edu.pk (S.K.); 2 Department of Geography, Hong Kong Baptist University, Hong Kong; mah.sajjad@hotmail.com; 3 Department of Geography, University of Karachi, Karachi 75270, Pakistan; saima-ku@uok.edu.pk (S.S.); jkazmi@usa.net (S.J.H.K.)

**Keywords:** I-SpaDE, spatial–temporal analysis, disease mapping, Dengue Fever, public health planning, Geographic Information Systems

## Abstract

The spatial–temporal assessment of vector diseases is imperative to design effective action plans and establish preventive strategies. Therefore, such assessments have potential public health planning-related implications. In this context, we here propose an *integrated spatial disease evaluation* (I-SpaDE) framework. The I-SpaDE integrates various techniques such as the *Kernel Density Estimation*, the *Optimized Hot Spot Analysis*, space–time assessment and prediction, and the *Geographically Weighted Regression* (GWR). It makes it possible to systematically assess the disease concentrations, patterns/trends, clustering, prediction dynamics, and spatially varying relationships between disease and different associated factors. To demonstrate the applicability and effectiveness of the I-SpaDE, we apply it in the second largest city of Pakistan, namely Lahore, using Dengue Fever (DF) during 2007–2016 as an example vector disease. The most significant clustering is evident during the years 2007–2008, 2010–2011, 2013, and 2016. Mostly, the clusters are found within the *city’s central functional area*. The prediction analysis shows an inclination of DF distribution from less to more urbanized areas. The results from the GWR show that among various socio-ecological factors, the temperature is the most significantly associated with the DF followed by vegetation and built-up area. While the results are important to understand the DF situation in the study area and have useful implications for public health planning, the proposed framework is flexible, replicable, and robust to be utilized in other similar regions, particularly in developing countries in the tropics and sub-tropics.

## 1. Introduction

Dengue Fever (DF) is a vector-borne disease with significant public health concerns in many tropical and subtropical countries as ~100–400 million cases are reported globally every year [1,2,3]. It is a neglected tropical disease and its more severe forms (i.e., dengue hemorrhagic fever (DHF) and dengue shock syndrome (DSS)) are now well evident as the leading causes of mortality and morbidity in different regions, with more concentrations in urban areas [4,5]. This disease affects poor/marginalized urban and peri-urban areas and communities, especially crowded neighborhoods. The female *Aedes Aegypti* and *Aedes Albopictus* mosquitoes are responsible for the transmission of the dengue virus (DENV) [6]. The DENV is circulated in five distinct serotypes ranging between DEN 1 and 5 [7,8]. The serotypes 1–4 follow humans and 5 follows a sylvatic cycle [9]. DF is geographically expanding and becoming a serious disease due to its socio-economic, health, and environmental correlations [10,11]. Compared to the previous 50 years, the recent global incidence of dengue has climbed to 30-fold [12,13,14], and the unavailability of a safe vaccine is further worsening the menace [15]. The World Health Organization (WHO) estimated ~390 million annual DF infections in 128 countries, placing ~3.9 billion people at risk [16,17,18,19].

Pakistan, a developing country in South Asia, has faced a rising trend of dengue prevalence since 1994 [20,21,22]. In 2019, 52,485 DF-positive cases and 91 deaths were reported nation-wide. For economically deprived nations that are relatively poorly equipped with health facilities, such as Pakistan, this epidemic has exerted a tremendous economic burden on provincial and federal governments, especially in Punjab province. About 20,980 cases were reported solely in two cities (i.e., Rawalpindi and Islamabad Capital Territory—40% of the overall national tally) [23,24]. Previously, disasters such as floods during 2010 also made the situation worse [25], when DF cases in Pakistan rose significantly from 4500 in 2005 to 21,204 in 2010 [26]. Approximately 96% of the cases were reported in urban areas [21] occurring mainly during the post-monsoon season [27]. Several factors played a considerable role in the evolution of dengue in Pakistan. These include the absence of any information platform, ineffective controlling plans, poor public health infrastructure, uncontrolled population growth, and rapid urbanization with deprived sanitation [28,29].

The existence of coinfections of dengue and COVID-19 also exhibits an alarming situation [30]. Such coinfections can lead towards a co-epidemic, which could be disastrous for the national health system [31]. Given this situation, the increasing risk of DF demands the attention of relevant authorities and public/private sectors in terms of applied and contemporary approaches to help in designing effective control strategies and eradication plans. Similarly, the country lacks in the adaptation of new technological solutions such as Geographic Information Systems (GIS) and various space–time pattern mining techniques to achieve solutions to vector disease-related challenges (prevention, control, and mitigation).

In disease studies, populations, places, and times are the fundamentals of disease outbreak investigations [32]. Therefore, to reduce/eradicate the DF-associated risks, it is imperative to improve the spatial and temporal understanding of such diseases so that appropriate plans could be designed in advance for future vigilance [33,34,35,36,37,38,39]. In this context, huge investments in the health sector are being made by national and sub-national governments [40,41,42]. However, developing inclusive as well as operative tools to tackle the issues of disease outbreaks, and their integration in local and national action plans, is very important and useful, as shown by the recent COVID-19 outbreak worldwide [43,44,45,46]. With the advancements in geo-data collection, the development of spatial distributional models, the introduction of data-driven interactive platforms, and the establishment as well as the integration of decision-support systems, there is a broader opportunity to formulate efficient, cost-effective, and robust tools and frameworks to analyze the spatial and temporal dynamics of vector diseases such as DF along with different driving factors behind the spread [47,48,49,50,51,52,53,54]. These integrated tools and frameworks could potentially help decision-making and resource allocation to take place more efficiently than the typical reductionist approaches—supporting the overall prevention and response efforts made at different administrative levels.

While geo-informatics has broadened our understanding of public health problems, spatial analytics facilitated by GIS are proven to help in addressing the modern needs of risk analyses. As a result, researchers in recent years have conducted an increasing number of studies related to geospatial perspectives of public and environmental health [29,55]. Several studies utilized spatial–temporal analyses for vector diseases (e.g., DF) risk modeling in different areas of the world [56,57,58,59,60,61,62,63,64]. While such studies are rare in many developing countries in South Asia (e.g., Pakistan and Bangladesh), few, if any, have presented a comprehensive framework to integrate various in-practice spatial models with advanced space–time pattern mining techniques to explore the spatial–temporal perspectives of vector diseases [65,66,67,68,69]. Most of the existing literature focuses on the application of individual geo-information models such as Hot Spot analysis. For instance, some Pakistan-based studies, such as Khalid and Ghaffar [70], Khan et al. [71], Khalique et al. [72], and Hafeez et al. [73], have used hot spot and other spatial autocorrelation (e.g., Local Indicator of Spatial Autocorrelation—LISA) analytics. However geo-integrative disease modelling has been neglected. These reductionist tools mostly focus on a single aspect of the data, which hinders comprehensive evaluation. For example, the simultaneous analysis of both spatial and temporal aspects of disease data is not well represented in the existing literature. Similarly, existing studies in disease evaluation lack the integration of the aforementioned typical tools with advanced and more recent spatiotemporal models such as space–time patterns mining analytics. Another neglected aspect is the integration of prediction simulations to aid pre-event decision-making. Therefore, integrating in-practice typical geo-information models with advanced analytics-based techniques can progressively provide in-depth insights related to diseases. In this context, this research fills the knowledge gap by integrating in-practice spatial analytical approaches with advanced space–time pattern mining analytics and prediction. This combined approach would be more effective as compared with the typical isolated approaches.

In light of the above, we present an *integrated spatial disease evaluation* (I-SpaDE) framework based on the coupling of spatial statistical distributional modelling and space–time pattern mining techniques. The proposed I-SpaDE framework employs advanced and contemporary approaches to evaluate and map the spatial–temporal dynamics of vector diseases (DF in this case). This is achieved by systematically integrating the spatial modelling techniques (i.e., *Global Moran’s I-based spatial autocorrelation and LISA*) with space–time pattern mining informatics (i.e., *space–time emerging hot spots analysis*). The space–time mining is also integrated with prediction analytics—making this framework a well-organized geo-analytical solution. To the best of our knowledge, the proposed framework is novel in its very nature in the context of spatial–temporal analysis of vector diseases and different associated factors. The study in hand provides insights regarding retrospective vector disease patterns (i.e., DF) and the possibilities for future space–time patterns of epidemics. Drawing on an illustrative case of DF in Pakistan’s second largest city, Lahore, we present the applicability of the proposed I-SpaDE framework. While the results from this study will have important implications for health-related decision-making and planning in Pakistan, the proposed framework could be equally useful for other global regions where vector diseases (e.g., malaria and DF) are common epidemics—subjective to a reasonable data understanding.

## 2. Materials and Methods

**Figure 1:**
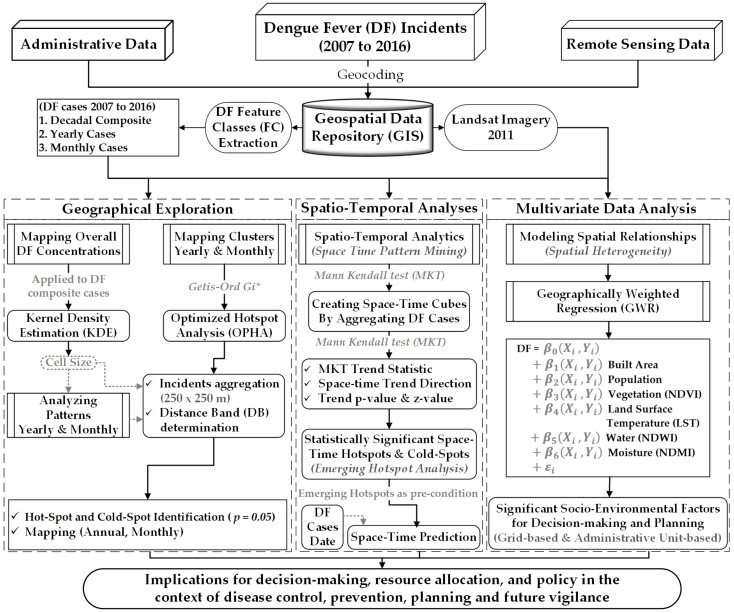
Methodological framework (I-SpaDE framework).

As aforementioned, the main focus of this study is to present the I-SpaDE framework. Within this framework (Figure 1), we first computed *Kernel Density Estimation (KDE)* by employing decadal point data, which assisted in figuring out the spatial cell size for further clustering analyses. Then, we analyzed spatial patterns of dengue cases through *Incremental Spatial Autocorrelation analysis* (ISA). This was achieved after splitting the data into yearly and monthly geo-layers. The ISA results (Supplementary Figures S1–S22 for annual and monthly, respectively) helped in the detection of further spatial hot and cold spots as it provides baseline bandwidths and thresholds for such inquires [74]. Thus, to achieve the statistical significance, we executed spatial local clustering statistics (i.e., *Optimized Hot Spot analysis*). Later, we explored the space–time patterns of dengue cases by adding a time (*t*) dimension, which is often overlooked in spatial disease assessments. The *space–time cube analysis* was employed for this spatiotemporal assessment to estimate the *Mann–Kendall Trend*, if any. Furthermore, the space–time hot spots were also mined through the *Emerging Hot Spot analysis*. In this specific way, these space–time analytics allowed the exploration of the integrative benefits of “Getis-Ord Gi*” statistics and *Mann–Kendall Trend* statistics. Furthermore, a prediction analysis was also carried out to discover “where could DF possibly emerge next?”. This prediction was also powered by space–time analytics, namely the *Emerging Hot Spot analysis*, as vital preconditions. Afterward, the association of DF with various socio-environmental/ecological factors was examined using different geo-analytical layers. The *Geographically Weighted Regression* (GWR) approach was employed for this assessment under the *2nd law of geography* that “*conditions change from place to place*”. ArcGIS 10.8 (ESRI: Redlands, CA) and ArcGIS Pro 2.8 (ESRI: Redlands, CA) were utilized for these spatial–temporal analyses. The framework furthered our understanding of such modelling integrations in analytical and predictive ways. It has also helped urban communities to design and establish a geographic corridor to effectively tackle such diseases’ epidemics. This unique modelling approach was applied to District Lahore, Pakistan, as shown in Section 2.1, and a detailed discussion of all aforementioned methods follows afterward (Section 2.2, Section 2.3, Section 2.4, Section 2.5 and Section 2.6).

### 2.1. Study Area

**Figure 2:**
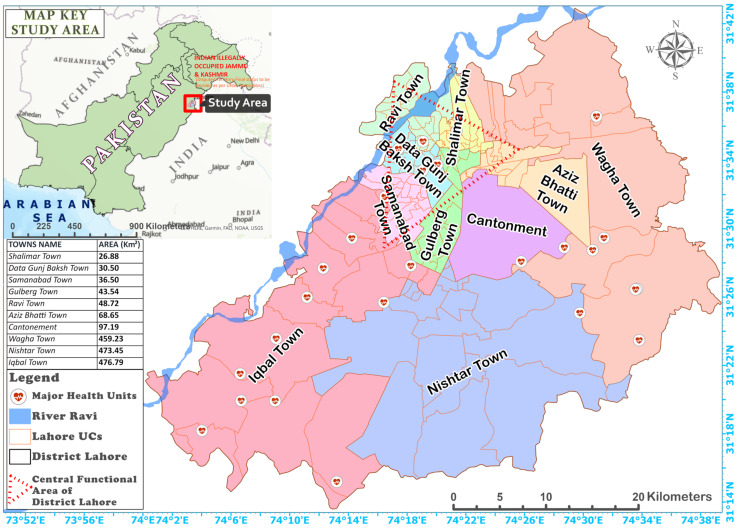
Study area map of city district of Lahore showing union councils (UC), towns, and district boundaries.

The application of the proposed I-SpaDE framework was demonstrated in district Lahore, Pakistan (area: 1761.46 km^2^; geographic coordinates: 31°25′52.57″ N, 74°21′31.49″ E; mean elevation from sea level: 217 m; Figure 2). The study area was divided into 150 Union Councils (UCs, local administrative unit in Pakistan) and one Cantonment area (relatively affluent areas). The *central functional area* (Figure 2) was demarcated based on town areas having a denser urban population, and greater land use and economic activity, because Lahore’s population is 83% urban and only 17% is rural, making it an urban district. Lahore is facing rapid urbanization where the population is growing at the rate of 3.2%. This rate is even larger than the average population increase in Pakistan, and hasty urban development is causing several issues such as temperature increases, which creates a suitable environment for dengue mosquitoes [28,75,76,77]. Naqvi et al. [28] explained climate and dengue synergism, reflecting the significance of climatic conditions for dengue transmission in Lahore. Heavy rainfall events in the late monsoon (mid-July to mid-September) occur every year in Lahore. The summer season begins in May and fades away in August with mean maximum and minimum temperatures of ~45.4 °C and ~29.3 °C, respectively. The Winter season starts in November and ends in February, with December–February being the coldest months with mean maximum and minimum temperatures of 21.1 °C and 7.2 °C, respectively. Sometimes, the minimum temperature in the winter drops to 1 °C [29,78,79,80,81,82]. These conditions aid dengue’s upsurge [29]. As Lahore has a favorable environment for dengue transmission, it is a good choice to demonstrate the applicability of the proposed framework. Additionally, Lahore was chosen because of its continuous epidemic situation since 2007, with the main outbreak in 2011 causing the death of 257 people [83,84].

### 2.2. Data Collection and Geo-Preprocessing

The laboratory-confirmed dengue cases data from 2007 to 2016 were collected from the Dengue Cell at the Directorate General Health Services Punjab (DGHSP at www.dghs.punjab.gov.pk/ (accessed on 6 January 2020)), Pakistan. Later, the data were geocoded into point objects where each point represented a dengue-confirmed patient. The final ten-point data featuring classes containing 19,349 cases were used for further integrated evaluation. The population and Built-up data (250 × 250 m^2^) for Lahore were acquired from the *European Commission’s Global Human Settlement* data portal for the period 1975–2015 (available at: www.ghsl.jrc.ec.europa.eu/ (accessed on 8 January 2020)). These datasets were used to obtain an overview regarding the process of urbanization during the past forty years, as well as a demarcation of the *central functioning area*. Moreover, the Landsat 5 (August 2011) imagery was downloaded from the *National Aeronautics and Space Administration* and the *United States Geological Survey* data portals. A geospatial data repository was established to store, manage, retrieve, and analyze the data.

The upcoming sections and subsections provide a comprehensive description regarding spatial and spatiotemporal methods used in this study:

### 2.3. Spatial Analyses

This section outlines the spatial analyses used in this study (i.e., Kernel Density Estimation (KDE) and Optimized Hot Spot analysis).

#### 2.3.1. KDE Analysis

The KDE can be applied to polyline or point features to compute a magnitude-per-area-unit employing a kernel function to fit a smoothly tapered surface. It calculates the magnitude/density of spatial features in a specific region/neighborhood around those features. This approach is widely used to detect and visualize patterns of different features [85,86,87]. In this study, the KDE analysis was performed using a combined point layer (2007–2016) to scrutinize areas with high density or magnitude of dengue cases. This analysis helped us to analyze the regions with relatively higher concentrations of DF cases in the study area and vice versa. One should note that the determination of spatial bandwidth and the selection of function are crucial because these factors control the visualized data. This study computed the bandwidth using the following:

(1) $$SearchRadius=0.9\times min\left(SD,\sqrt{\frac{1}{\ln\;\left(2\right)}}\times Dm\right)\times n^{-0.2}$$

where *SD* is Standard Distance, *Dm* is median distance and *n* is the sum of population field values [88]. As per the resultant value, the bandwidth was set to 790 m.

The results of the KDE were also supported by a new cartographic method known as the *Bivariate colors* (geo-association), which helped to visualize the quantities through bivariate colors based on two variables simultaneously. This unique technique emphasizes the highest to lowest values in the input data to identify the salient correlations [89]. Firstly, we calculated the population density in Lahore using the data at the UC level. Later, DF prevalence (*DFP*) was estimated using the following equation as suggested by Bonita et al. [90]):

(2) $$DFP=\frac{Total\;number\;of\;Dengue\;cases\;\left(UC\;wise\right)\;during\;2007–2016}{\;UC\;wise\;Population\;of\;Lahore}\times 100,000$$

Using this *DFP* and UC-level population density, we produced the bivariate map to explore their relationships. We here recognized the spatial uncertainty that could be observed by utilizing UC-level administrative boundaries for the representation of this bivariate relationship. Due to this, these aggregated cases to administrative zones were subject to *modifiable areal unit problem (MAUP)*. However, the purpose here was exploratory evaluation intended to guide towards targeted interventions [91]. Henceforth to deal with *MAUP*, we used original geocoded point data of dengue cases rather than aggregated ones [92]. Such spatial clustering statistics are also supported by valid *incremental spatial autocorrelation* methods to ensure unbiased scales by computing the bandwidth and threshold values.

#### 2.3.2. The Optimized Hot Spot Analysis (Yearly and Monthly)

The *Optimized Hot Spot Analysis* (OPHA) was utilized to pinpoint the regions with statistically significant clustering of DF incidents. This method computes the “Getis-Ord Gi*” statistic for each feature (dengue incident) and results in associated *z-scores* and *p*-values [93,94]. These scores highlight where features with either low or high values spatially cluster. This local statistic looks at each feature concerning its neighboring features. If a feature and its neighboring features possess high value, that particular feature would be a statistically significant hot spot. Then the sum of all features is compared with the local sum of a particular feature and its neighboring features. The Hot Spot analysis is applied using:

(3) $$G_{i}^{*}=\frac{\sum_{j=1}^{n}w_{i,j}x_{j}-\bar{X\;}\sum_{j=1}^{n}w_{i,j}}{S\sqrt{\frac{\left[n\sum_{j=1}^{n}w_{i,j}^{2}-{\left(\sum_{i=1}^{n}w_{i,j}\right)}^{2}\;\right]}{n-1}}}$$

where *x_j_* is the attribute value for feature *j*, *w_i_*,*_j_* is the spatial weight between feature *i* and *j*, and *n* represents the total number of features. $\bar{X\;}$ and S are computed using the following:

(4) $$\bar{X\;}=\frac{\sum_{j=1}^{n}x_{j}}{n}$$

(5) $$S=\sqrt{\frac{\sum_{j=1}^{n}x_{j}^{2}}{n}-{\left(\bar{X}\right)}^{2}}$$

The Gi* statistic returns the associated *z-scores.* The output *z-score* values > 2.58, 1.96 to 2.58, and 1.65 to 1.96 represent the statistically significant hot spots (clustering of higher values) with 99%, 95%, and 90% confidence levels, respectively. Spatial randomness ranges from −1.65 to 1.65 *z-score* values. Conversely, the output *z-score* values < −2.58, −1.96 to −2.58, and −1.65 to −1.96 represent the statistically significant cold spots (clustering of lower values) with 99%, 95%, and 90% confidence levels, respectively [12,95,96,97]. The OPHA uses the incident data aggregation method to aggregate the incident results in the fishnet or hexagon grids. Here, we used fishnet with a 250 m cell size. This cell size was computed in the light of KDE by dividing the longest side of the zone with the most intense areal density by 100 (i.e., 25,026/100 = 250.26, unit m). The space–time analyses also employed the same cell size as the space–time distance intervals [88].

### 2.4. Space–Time Cube Analysis

**Figure 3:**
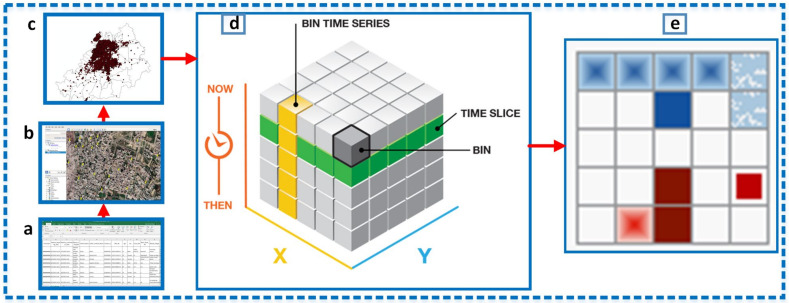
Towards space–time cube aggregation and *Emerging Hot Spot Analysis* (adapted from http://ESRI.com (accessed on 12 June 2020)): (**a**) Ten Excel sheets contain dengue cases from 2007–2016. (**b**) Geocoding in Google Earth and KML creation for all years. (**c**) KML to feature-classes conversion (2007–2016) and record joining. (**d**) Creation of *space–time cubes* by aggregating points, where points are counted within each bin and the space–time trend is calculated. (**e**) *Space–time cube* layers are used for the *Emerging Hot Spot Analysis*.

The KDE and hot spot analyses evaluated spatial patterns of DF incidents’ density, but they did not exhibit the time pattern characteristics of these occurrences. Hence, for the space–time pattern mining of such incidents, the *Space–time Cube analysis*, and the *Emerging Hot Spot analysis* were utilized (Figure 3), which are relatively new tools that are primarily applied to crime incidents and traffic accidents [88,98,99]. Hot spot analysis involves only the Getis-Ord Gi* statistic; however, we present a combined advancement in this space–time analyses which integrates the Getis-Ord Gi* statistic with the *Mann–Kendall test (MKT)* trend test. It is noted (to the best of our knowledge) that there has not been any application of these tools to study epidemics of vector-borne diseases (i.e., DF) in integration with spatial models. We argue that the integration of these tools with other spatial models (described in Section 2.3) could progressively help in the exploration of vector diseases, which might have important implications in disease control planning and decision-making as well as in relation to health challenges.

A *space–time cube* binds space–time in a three-dimensional (3D) data structure called a netCDF (*Network Common Data Form*), which comprises a 3D array of bins showing the absolute location (x–y-dimensions) and absolute time (z-dimension) simultaneously. These bins divide the whole study area into equally defined 3D grids. Within each grid, points are counted with their respective time [100]. In this way, we aggregated the dengue incidents within 250 × 250 m^2^ (distance interval) fishnet grids with an absolute time step interval of 1-week using the end-time aggregation method, where one continuous horizontal and vertical bin composite showed the time slice and the bin time series, respectively (Figure 3d). This approach allowed the investigation of a vector-borne disease phenomenon (i.e., DF) in the context of “where” and “when”. For trend calculation, this approach used the well-known *Mann–Kendall trend test*, which helped in determining the respective trend for bin values (counted points) across time at each absolute location.

As a non-parametric test, the *Mann–Kendall test (MKT)* can detect statistically significant trends, if any, in the time series of a given incident such as the DF cases in this study [101,102,103,104,105,106,107]. The MKT extends the capabilities of a space–time cube by measuring the monotonic or non-monotonic trend across time within each bin of the cube. It is executed on every location as an independent bin time-series test. It analyzes bin values/counts and time sequences using *rank correlation statistics*. The null hypothesis *H*_0_ assumes that the data does not follow any monotonic trend while the alternative hypothesis (*H*_1_) aims to reject the *H*_0_, and implies that the data follow a monotonic trend (either upward or downward). These bin counts for the first and second time-periods are compared below [100,108]:

(a) First time period bin value < Second time period bin value = +1;
(b) First time period bin value > Second time period bin value = −1;
(c) Both values are same = 0.

After the comparison of the results for each period, their sum is expected to be zero, indicating the absence of any trend in the counts over time. Overall variance in the results of bin count series is compared to expected zero-sum values to observe the statistically significant differences. The trend of every time series is represented as the *z-* and *p*-value, where smaller *p*-*values* show a higher significance of the trend. Furthermore, positive and negative *z-scores* display an increase and/or decrease in bin counts, respectively. The statistic of the *Mann–Kendall test* can be calculated as follows: [109,110,111].

(6) $$S=\overset{n-1}{\underset{k=1}{\sum }}\overset{n}{\underset{j=k+1}{\sum }}sgn\;\left(X_{j}-X_{k}\right)$$

where:

(7) $$sgn\left(x\right)=\left\{\begin{array}{c} 1\;if\;x>0\\ 0\;if\;x=0\\ -1\;if\;x<0 \end{array}\right.$$

The mean of *S* is *E[S]* = 0 and the variance is:

(8) $$\sigma^{2}=\left\{n\left(n-1\right)\left(2n+5\right)-\overset{p}{\underset{j=1}{\sum }}t_{j}\left(t_{j}-1\right)\left(2t_{j}+5\right)\right\}/18$$

The *Z*-transformation is calculated as:

(9) $$Z=\left\{\begin{array}{c} \frac{S-1}{\sigma }\;if\;S>0\\ 0\;\;\;\;\;\;\;\;\;if\;S=0\\ \frac{S+1}{\sigma }\;if\;S>0 \end{array}\right.$$

The statistic *S* is closely related to Kendall’s *τ* as given by:

(10) $$\tau =\frac{S}{D}$$

where *D* represents:

(11) $$D={\left[\frac{1}{2}n\left(n-1\right)-\frac{1}{2}\overset{p}{\underset{j=1}{\sum }}t_{j}\left(t_{j}-1\right)\right]}^{\frac{1}{2}}\;{\left[\frac{1}{2}n\left(n-1\right)\right]}^{\frac{1}{2}}$$

This was applied to annual dengue cases between 2007 and 2016, and the results are presented as a table showing the trend direction, *z-score*, *p*-value, and remarks regarding *H*_0_ (Hypothesis acceptance) and *H*_1_ (Hypothesis rejection).

#### Measuring Emerging Hot Spots

This approach is based on the *Emerging Hot Spot Analysis* in ArcGIS and is known to be effective in identifying space–time clusters (Figure 3e). It uses a *3D space–time cube* as an input and displays spatial–temporal hot spot trends in *2D geo-visualization*, if any. It starts from a conceptualization of the spatial relationships using the provided values to compute the Getis-Ord Gi* statistic, or from a hot spot analysis for each bin of the study area. The analysis results in space–time hot spot *z-score* and *p*-values associated with each bin. Afterward, the application of the *Mann–Kendall trend test* helps to evaluate the hot spot and cold spot trends. Hence, with hot spot and cold spot *z-scores* and *p*-values for each bin and a trend *z-score* and *p*-value for each location, the *Emerging Hot Spot Analysis* categorizes the space–time trending hot spots into seventeen different types including new, consecutive, sporadic, and oscillating hot/cold spots [88,112,113,114,115]. These results are mapped in 2D using a 250 × 250 m^2^ grid for the purpose of communication.

Here, one might notice that all the methods present an integrative performance. They not only improve themselves but also provide feedback to other superior models such as KDE, which helps in cell size calculation and spatial hot spot analysis and paves the way to space–time clustering. This modelling interrelationship is neglected in disease studies, especially in developing countries. This methodology may add new insights to the spatial modelling of vector diseases, and could progressively help in decision making, resource allocation, and policymaking—supporting overall prevention, planning, and future vigilance.

### 2.5. Modeling the Space–Time Prediction Zones

**Figure 4:**
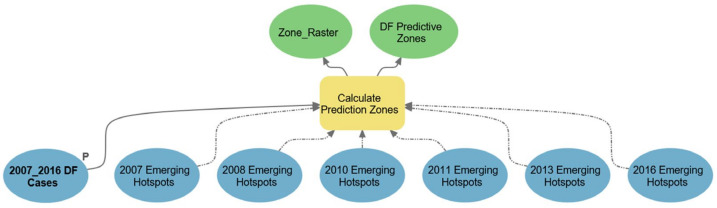
Model showing DF prediction process where one major parameter (P) is DF composite cases. A total of six precondition layers (DF significant space–time hot spots 2007–2016) were also joined. After devising unique spatiotemporal ranges, predictive risk locations were calculated as vector and raster layers.

Space–time analyses are significant but if the prediction is added, it becomes more useful for decisions and policies regarding disease prevention and control. For this purpose, we utilized a unique approach that helps to identify areas/zones at risk of near-repeat and repeat DF incidents by setting the specific spatial–temporal ranges of DF influence of past incidents. We used a point-feature class of DF cases. Some precondition layers encompassing DF *emerging hot spots* (2007–2016) were also joined with the model. Later, the initial processing date was set to 3/8/2016. The spatial range of influence and spatial half-distance were set to 4695.23 m and 2347.615 m, respectively. The model looked into the future by utilizing post (temporal range of influence: 130 days) and prior temporal ranges (temporal half-life: 65 days) to the initial processing date, and cumulatively predicting DF risk. The movements up and down towards the initial date determined increasing and decreasing spatial–temporal influence, respectively (Figure 4). The predictive zones were mapped as 5 major categories denoting the Highest, High, Moderate, Low, and Lowest risk areas. This technique was devised in ArcGIS Pro, and is primarily used for crime spatial–temporal point-pattern prediction. However, we believe that the approach is equally useful for other incidents with a reasonable understanding of the data. Therefore, we devised it for vector diseases (DF in this study) with some additional time hot spot and cold spot pre-conditions. The output of this modeling approach may be better than other parallel predictive methods [116].

### 2.6. Evaluating Different Spatial Socio-Environmental Factors of DF: A Multivariate Analysis

It is well documented that the emergence and outbreak of vector diseases are associated with several socio-environmental/ecological factors, which can act as a stimulus [117]. The same is the case with DF as different driving forces within the urban built-environment might provide suitable conditions for DF to emerge/spread [118,119]. Evaluating these factors is of high importance to identify the outbreak situations even before they might take place. This evaluation can also help in the design and planning of urban built environments in terms of ensuring more resilience in the wake of vector diseases [120]. Usually, the *Ordinary Least Squares* (OLS) regression—a global regression modelling approach—is used to identify the association of vector diseases with its driving factors. However, the absence of spatial elements in OLS might not be that useful in the context of geographical distributional assessments such as the one in this study [121]. Therefore, to explore the association between different potential factors and DF, we used the *Geographically Weighted Regression* (GWR) technique [122]. This technique has an upper hand as compared with OLS because it does account for the spatial element while fitting the model. Additionally, it outperforms OLS as the results from OLS are usually unreliable if there is multi-collinearity among the predictors [123,124].

In the GWR method, local equations are built for each feature of the dataset by incorporating the dependent and explanatory variables. Based on the existing literature, we compiled a list of six potential predictors to be used for the GWR model (Table 1).

These predictors included built-up area (explained by the *Normalized Difference Built-up Index*—NDBI), population, vegetation (explained by the *Normalized Difference Vegetation Index*—NDVI), land surface temperature (LST), water (explained by the *Normalized Difference Water Index*—NDWI), and moisture (explained by the *Normalized Difference Moisture Index*) [126,127,140,141,142,143,144]. It is noted that the indices utilized to represent the environmental factors are well established in the literature and have been extensively used to study several environmental problems. Before fitting the model, we computed the *variance inflation factor (VIF)* to check the multi-collinearity among the selected variables. As per the rule of thumb, if the *VIF* value of a certain variable was >7.5, it was excluded from the model [145,146,147,148]. In this study, we used a leave-one-out procedure and performed several iterations until there was no multi-collinearity among the variables. To utilize the DF incidents as the dependent variable, we used the KDE-based density estimation. The following model was proposed to explore the relationship between the independent and explanatory variables, if any:

(12) $$\mathrm{DF}=\beta_{0}\left(X_{i}\;,\;Y_{i}\right)\;+\;\beta_{1}\left(X_{i}\;,\;Y_{i}\right)\;\mathrm{Built}\text{-}\mathrm{up}\;\mathrm{Area}\;(\mathrm{NDBI})\;+\beta_{2}\left(X_{i}\;,\;Y_{i}\right)\;\mathrm{Population}\;+\beta_{3}\left(X_{i}\;,\;Y_{i}\right)\;\mathrm{Vegetation}\;(\mathrm{NDVI})\;+\beta_{4}\left(X_{i}\;,\;Y_{i}\right)\;\mathrm{Land}\;\mathrm{Surface}\;\mathrm{Temperature}\;(\mathrm{LST})\;+\beta_{5}\left(X_{i}\;,\;Y_{i}\right)\;\mathrm{Water}\;(\mathrm{NDWI})\;+\beta_{6}\left(X_{i}\;,\;Y_{i}\right)\;\mathrm{Moisture}\;(\mathrm{NDMI})\;+\varepsilon_{i}$$

where $\varepsilon_{i}$ is the error term [149].

## 3. Results

### 3.1. Exploring DF Frequencies on an Annual and Monthly Basis

**Figure 5:**
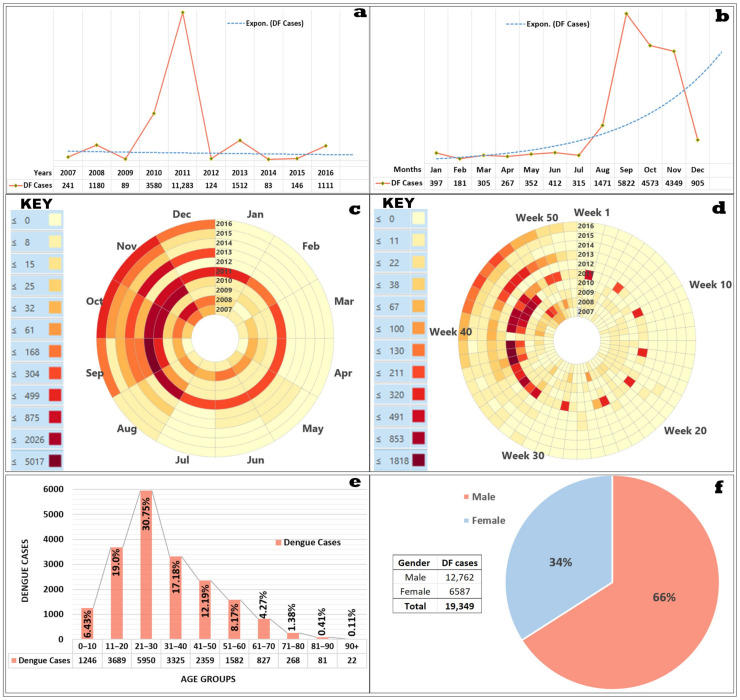
(**a**) Annual dengue epidemic curve (2007–2016), (**b**) monthly epidemic curve (2007–2016), (**c**) monthly dengue data clock showing dengue case date counts by months over years, (**d**) weekly dengue data clock showing dengue cases date counts by weeks over years, (data clocks: 2007 to 2016; innermost ring to outermost, respectively), (**e**) dengue-affected age groups, (**f**) dengue-affected genders.

DF remained a dreadful disease during the decadal periods (2007–2016) due to repeated infection aggravations as illustrated in Figure 5a–d. A total of 19,349 confirmed DF cases were reported, with a higher concentration between 2010 and 2011 (76.81%). Other than these years, DF showed considerable cases (≥1000) only during 2008, 2013, and 2016. The DF risk mainly remained highest in August–November (16,215 cases). These months and their respective weeks were predominantly affected (Figure 5b–d). Within this affected population, males (66%) and adult age groups ranging from 21 to 30 years old (30.75%) experienced the highest number of infections (Figure 5e–f). These results are in agreement with a previous study in Khyber Pakhtunkhwa, Pakistan, by Abdullah et al. [150]. Convincingly, adult males are more susceptible to mosquito (predominantly *Aedes aegypti*) bites than females. This is because adult males are more involved in outdoor activities, such as business and frequent travelling. Such mobilities are less common among the female population due to cultural settings and household restrictions [29,151].

### 3.2. Spatial Characterization of DF Incidents: Kernel Density Estimation (KDE) Analysis

**Figure 6:**
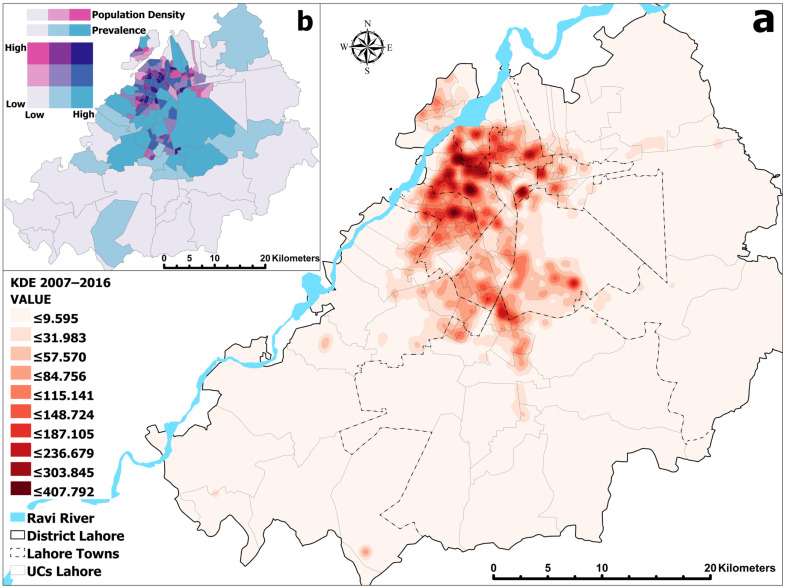
(**a**) Kernel Density Estimation showing the magnitude of dengue cases (2007–2016), (**b**) bivariate correlations at UC level, between Lahore Population Density and DF Prevalence (2007–2016).

Figure 6a,b demonstrate that DF cases mainly occurred where the population density in the study area was higher. The highest concentration of DF incidents was within the *central functional area* in Lahore. The major affected towns were *Data Gunj Baksh*, *Samanabad*, and neighboring towns such as *Shalimar*, *Ravi*, *Aziz Bhatti*, *Gulberg*, and the *Cantonment area*. Some clusters were also observed in the peri-urban neighborhoods of *Allama Iqbal town* and *Nishtar town* (Figure 6a). Overall, the urban UCs, with a total population of ~9 million, observed 17,493 infections (>90%). In these UCs, the minimum, maximum, mean, and standard deviation (SD) of *DFP* remained 18,673, 190.5 and, 126, respectively. The bivariate relationship between *DFP* (dependent variable) and population density (independent variable) was the highest in these areas, revealing the endemic foci during the study period (Figure 6b). Many studies, such as that of Naqvi et al. [28], affirm that dengue has evolved as a predominantly urban disease with urban mosquitoes, (i.e., *Aedes aegypti*). The outbreaks in Lahore were greatly enhanced by variable climatic effects, a lack of vector control, a lack of public health facilities, and uncontrolled urbanization, and population upsurges. Various other factors were related to community inadequacies such as poor water supply management, which forced urban dwellers to store water in their own containers, which could be left open. The education or awareness status of the public and poor sanitary conditions further added to the menace.

### 3.3. Detection of Hot Spots and Cold Spots

#### 3.3.1. Annual Assessment

**Figure 7:**
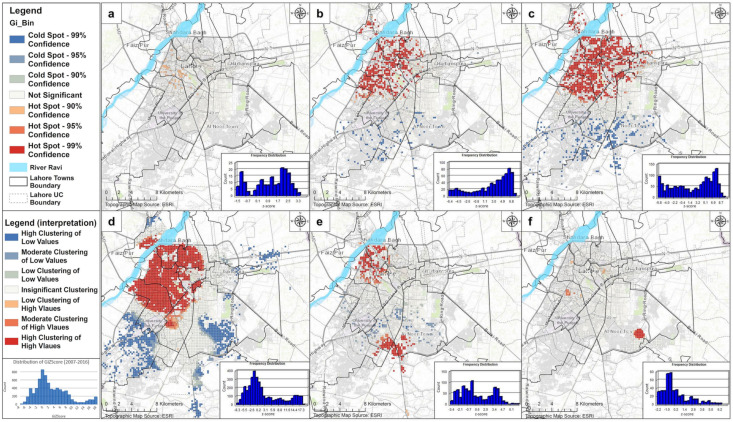
The *Optimized Hot Spot Analysis* showing dengue’s annual (2007–2016) statistically significant hot- and cold spots: (**a**) 2007, (**b**) 2008, (**c**) 2010, (**d**) 2011, (**e**) 2013, (**f**) 2016.

Based on the *Optimized Hot spot Analysis* tool and using the distance thresholds from annual ISA outputs (Supplementary Figures S1–S10), the statistically significant hot spots and cold spots were found in 2007, 2008, 2010, 2011, 2013, and 2016. Other years such as 2009, 2012, 2014, and 2015 experienced *complete spatial randomness* (CSR). Figure 7 and Supplementary Table S1 illustrate a clear picture of DF clustering in the significant years. In 2007, DF started with the lowest effects in the major towns (i.e., *Data Gunj Baksh* and *Samanabad*), which turned into a major concern during 2008 when DF spread out to almost all parts of the *central functional area*. Then, in 2010, DF agglomerated in that area even further and turned into a disastrous disease (in terms of morbidity and mortality) in 2011 when clustering achieved its peak in several major parts of the *central functional area*. In 2013, the agglomeration considerably decreased in the *central functional area* (mainly remaining in *Data Gunj Baksh* town and lower parts of *Ravi town*); however, it emerged in new areas located near/at the borderlines of *Gulberg town*, *Shalimar town*, *Allama Iqbal town*, and the *Cantonment area*. From there, it shifted to the *Cantonment area* during 2016. Hence, DF remained prevalent mainly in the *central functional area* during 2007, 2008, 2010, and 2011. However, in 2013, those clusters were weakened and divided into two distinct parts; one remained (lower agglomeration) in the *central functional area* and the other moved towards moderately urban and peri-urban areas. In 2016, clustering in the *central functional area* was at its lowest. Only one main cluster with an area of 3.62 km Sq. appeared within the lower *Cantonment area* nearby the moderately urbanized UCs. The shifting of these clusters to affluent areas is interesting and may be attributable to the lawns available in affluent areas where stagnant fresh water in plant-pots may have helped to increase the ratio of *Aedes* mosquitoes. A review by Bostan et al. [152] revealed that the presence of open swimming pools in these affluent localities provided suitable habitats for dengue mosquitoes. Another more pronounced reason may be linked to frequent work-related travelling from poorer areas to these localities [153].

#### 3.3.2. Monthly Assessment

**Figure 8:**
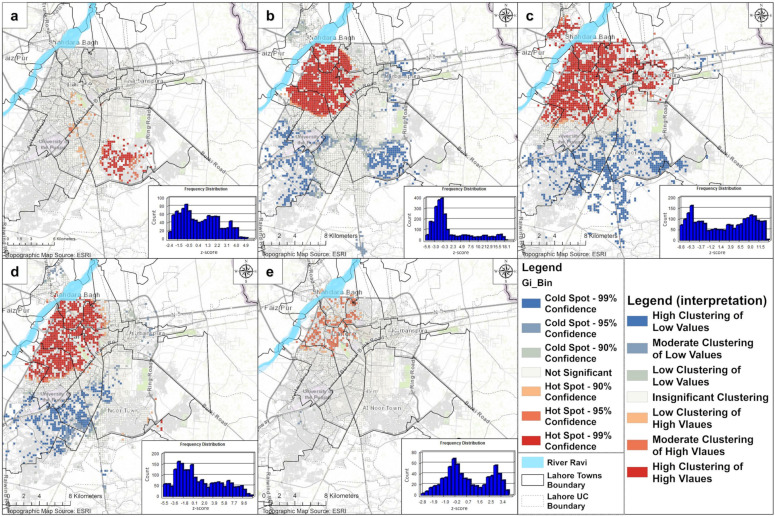
The *Optimized Hot Spot Analysis* showing dengue’s monthly (2007–2016) statistically significant hot spots and cold spots: (**a**) August, (**b**) September, (**c**) October, (**d**) November, (**e**) December.

Based on the *Optimized Hot Spot Analysis* tool and using the distance thresholds from monthly ISA outputs (Supplementary Figures S11–S22), it was found that the hot spots mainly emerged during August to December. Earlier months in the study years (January–July) were not identified as the statistically significant hot and cold spots. Figure 8 and Supplementary Table S2 show that significant months had clustering variability. In August, major statistically significant hot spots were identified, which were located inside the *Cantonment area*. However, the *central functional area* had minute clustering. In September, massive clustering happened in the *central functional area* with the highest *z-scores* and agglomeration (mainly in *Data Gunj Baksh town*, *Samanabad town*, *Shalimar town*, and some adjacent parts of *Ravi town* and *Gulberg town*). In the next month, clustering expanded from the *central functional area* with relatively lower *z-scores* to new locations such as *Allama Iqbal town*, *Ravi town*, *Aziz Bhatti town*, *Wahga town*, and the *Cantonment area*. During November, clustering shrunk back mainly to the *central functional area* and started diminishing in December.

### 3.4. Spatial–Temporal Evaluations

#### 3.4.1. Space–Time Cube-Based Mann–Kendall Trend (MKT)

The results from the MKT trend analysis show that the overall DF incidents exhibited increasing monotonic trends in each year except 2009. From 2007 to 2008, the *z-score* trend increased from 2.75 to 3.55 (Table 2). Afterward, the analysis showed its first peak at a *z-score* of 5.15 in 2010, unveiling it as the second most active year when DF cases occurred mostly from August to November. Although 2011 was spatially the most active year, it was not the most active year spatiotemporally because of its wide distribution of DF cases throughout the year. The year 2012 was also spatiotemporally positive with the lowest *z-score* (2.14) due to having most of the DF cases within September to October. The most spatiotemporally active year was identified to be 2013 when a high number of DF cases occurred from October to December (z-score: 5.58). During the next years (2014–2016), 2014 had the highest trend (4.20), which lessened in 2015 and 2016 (2.82 and 2.59, respectively). Although there were lesser numbers of DF cases in 2014–2015, higher trends emerged due to the closer spatiotemporal proximity (September–October in 2014 and October–November in 2015). In 2016, DF cases increased relatively, which were also distributed mainly between September and December. This represented a relatively lower but significant trend.

#### 3.4.2. Spatiotemporal Hot Spot Detection: Emerging Hot Spot Analysis

**Figure 9:**
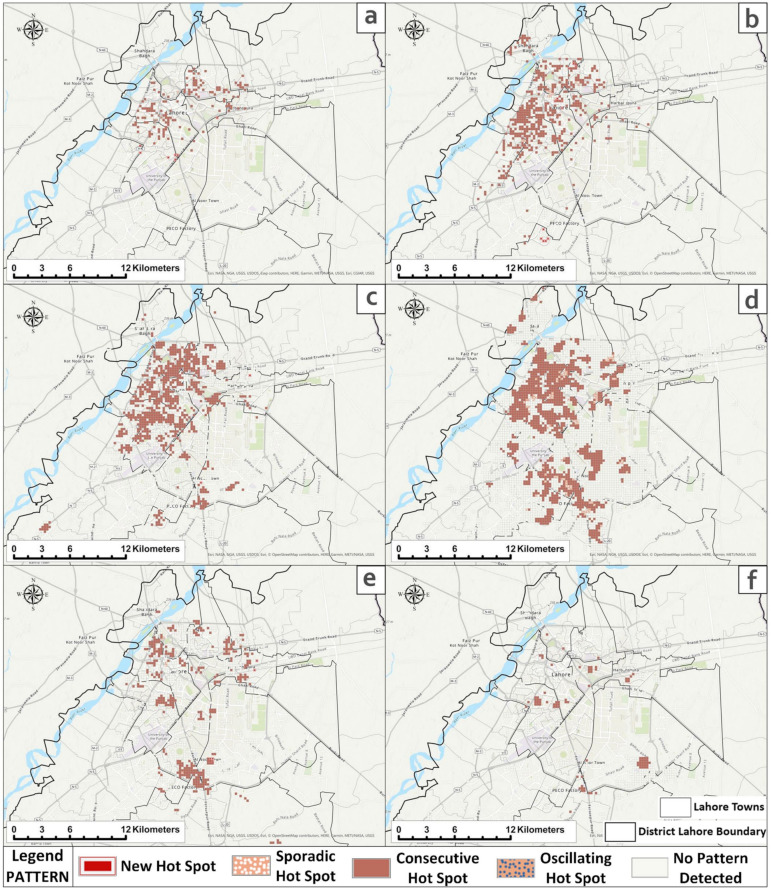
Space–time hot spots of DF using the *Emerging Hot Spot Analysis*: (**a**) 2007, (**b**) 2008, (**c**) 2010, (**d**) 2011, (**e**) 2013, (**f**) 2016.

The results show that the significant trending clusters emerged only during 2007, 2008, 2010, 2011, 2013, and 2016. Other years such as 2009, 2012, 2014, and 2015 did not exhibit space–time clustering. The *central functional area* was the major affected area throughout the decade. In 2007, there were 5 new, 141 consecutive, and 6 oscillating hot spots mainly located in the *central functional area*. In the same area, during 2008, clustering advanced with 13 new, 444 consecutive, and 4 sporadic hot spots. During 2010, 2 new and 730 consecutive hot spots emerged, mainly in the *central functional area* with some more extension towards *Gulberg town*, *Iqbal town* (*UC#121*), *Nishtar town* (*UC#138–140*), and the *Cantonment area*. During 2011, consecutive (total 1095) and sporadic (total 92) hot spots were increased substantially in the *central functional area*. Some exceptions were also identified such as the ones located in *Gulberg town* (around *Model town* and *UC#97–98*), *Nishtar town* (*UC#134–137* and *141–143*), and *Iqbal town* (*UC#138–140*). In 2013, this clustering decreased to 5 new (*Cantonment* and *Aziz Bhatti town*), 7 sporadic, and 322 consecutive hot spots (mainly in the *central functional area* and the *peri-urban* areas located at the borderline of *Gulberg*, *Nishtar*, and the *Cantonment area*). During 2016, a total of 85 consecutive hot spots emerged. The locations were similar to 2013; however, the clustering intensity was not the same. One major difference was the agglomeration inside the *Cantonment area* reflecting that DF could also emerge in affluent areas. In a nutshell, the *central functional area* in the study area was identified as the most vulnerable region to DF in terms of its spatial–temporal aspects. This situation calls for the prioritization of this area for special measures during the active months of DF in the study area (Figure 9).

#### 3.4.3. Space–Time Prediction

**Figure 10:**
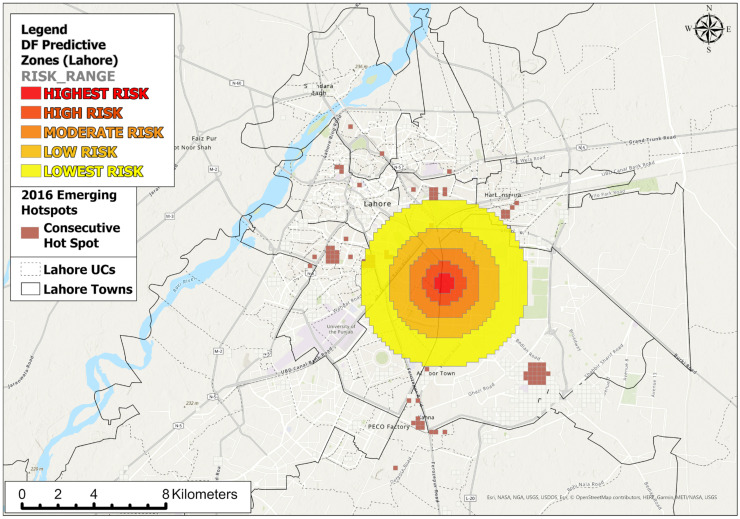
DF prediction zones based on repeat and near-repeat incidents that follow a spatiotemporal influential range of previous DF events.

Based on the current ((initial date when the first DF case occurred—2016—onwards; gradually increasing importance)) and previous ((2015–2007: gradually decreasing in importance with each earlier year)) DF cases and space–time clusters, the future DF zones were simulated, and these are illustrated in Figure 10. The prediction suggested that DF could have more tendency to occur within both *Gulberg town* and the *Cantonment area* simultaneously. It showed that the risk to the *Central Functional area* could be minimal. These zones of highest-to-moderate risk could be the next endemic foci, where measures through effective decision-making, appropriate actions, and operative policies are urgently required.

### 3.5. Association between Socio-Environmental Factors and DF

We present the association of DF and different socio-environmental/ecological factors at two different scales. including administrative unit-based (UCs in this case) and grid-based (250 × 250 m^2^) assessment. While the administrative unit-based analysis is useful to inform the local governments about the situation, the grid-based assessment is important to evaluate the relationships between DF and different factors at granular levels. We noted a multi-collinearity among the explanatory variables and used a leave-one-out approach to obtain the explanatory variables without the multi-collinearity. While this approach limits the ability of the model to some extent in the context of having more information on different factors, it is a rigorous technique, which is recommended to comprehend the issues related to multi-collinearity. After addressing the multi-collinearity, our final model included three factors out of a total of six (i.e., NDBI, NDVI, and LST).

The GWR model for the grid-based analysis resulted in an adjusted R^2^ of 0.84, showing that the model explained ~85% of the variance (Table 3). On the other hand, the adjusted R^2^ value for the administrative unit-based model was 0.73. This situation showed that the grid-based model, when used to explore the association between DF and different factors, outperformed the administrative unit-based model. However, the *Akaike information criterion (AIC)*, a well-known parameter to compare the model performances, indicated that the administrative unit-based model performed much better than the grid-based model as the difference between the *AIC* values was much larger than the generally accepted cutoff value (i.e., 3 or above).

Both the models showed that the relationship between LST and DF was the strongest among all three factors (mean βs values 27.790 and 20.529 for grid- and administrative unit-based models, respectively). The NDVI was the second most associated factor followed by the NBDI. This shows that the temperature was the most significant factor of DF in the study area, followed by the vegetation. The built-up area was the least associated. However, as the built-up area is also known as the major driving factor for LST and vegetation in urban regions, its importance cannot be undermined in the context of health planning in cities.

**Figure 11:**
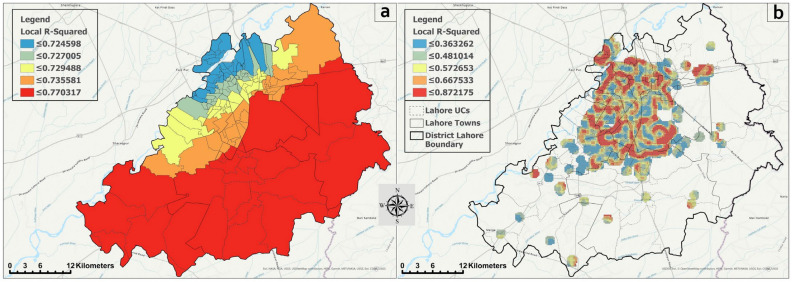
Spatial distribution of local R^2^ values for (**a**) administrative unit-based and (**b**) grid-based models.

The spatial distribution of the local R^2^ for both models (i.e., grid-based and administrative unit-based) is presented in Figure 11a,b. This distribution showed the fitness of the model for each unit of analysis (i.e., UCs and grids in Figure 11a,b, respectively). In the case of the administrative unit-based model, there was a little spatial variation in the performance of the model, as evident from the smaller range of the local R^2^ (0.72 to 0.77; Figure 11a). However, it was notable that the model performed relatively better in the more urbanized UCs (blue color), and this performance decreased when moving towards the outskirts of the study area (red shade)—presenting a clear geographic pattern. On the contrary, the grid-based model had a relatively wider range of local R^2^ values (0.36 to 0.87), showing a larger variability in the performance of the model (Figure 11b). There was no clear spatial pattern in the performance as one can see the mix of higher and lower R^2^ values throughout the study area. However, an inclined significance can be seen nearby *Cantonment area* locations where the dengue pattern shifted during 2016.

## 4. Discussion

DF is considered endemic in Pakistan due to its continuous emergence and reemergence during the last 30 years [28,154,155]. It has socio-economic/demographic and environmental concerns and the vital role of socio-economic as well as environmental factors in DF’s increase cannot be denied [156,157]. Fundamental risk assessments are crucial for the preventive planning and control of DF. Considering the complexity and reemergence of dengue, a comprehensive geospatial risk analyses framework is inevitable. Thus, we presented a systematic I-SpaDE framework that integrates various important geo-analytics such as spatial–temporal techniques as well as predictive and heterogeneity-based models. It is noteworthy that the integration of GIS-based space–time analytics, spatial distributional models, and detailed DF retrospective data provides important insights regarding the spatiotemporal DF risk within an urban area [95,158]. The importance of such tools/frameworks has become highly visible and evident during the current global COVID-19 emergency [159,160,161]. It is disastrous that DF risk is coupled with COVID-19 and the risk of coinfections could be higher during DF’s most favorable months (August–November/monsoon and post-monsoon) within urban and peri-urban areas or even rural neighborhoods [28,162,163,164]. Several coinfections have already been reported from various Asian countries including Singapore, Thailand, India, Bangladesh, and Pakistan [30,165]. It is feared that such coinfections could lead to co-epidemics [162,166]. In this scenario, the present study was employed to investigate the drastic DF situation in Lahore (study area), where a co-epidemic could exert tremendous pressure on an already struggling health system.

The proposed framework could be a practical, cost-effective, and robust tool to cope with DF epidemics in urban environments of tropical and sub-tropical countries. The framework follows spatial–temporal perspectives, which provide answers to “where” and “when” in the context of public health planning. It further makes it possible to comprehend well-known and operative methods from a single geodatabase repository. This concurrent approach helps in analyzing patterns, mapping clusters, and mining space–time patterns, supports the prediction of space–time risks, and the modeling of spatially varying relationships between vector diseases and their potential socio-environmental factors, as detailed in Section 3.

The primary focus of the I-SpaDE framework is the identification and understanding of vector diseases (DF in our case) in space and time, the exploration of its patterns/trends, and the identification of statistically significant clusters, which is considered one of the fundamental measures for effective surveillance and control of vector diseases [167]. Therefore, the proposed framework is important for public health authorities to better evaluate and understand the spread of DF along with its factors. From a public health perspective, the results are potentially important for the professionals to analyze the situation regarding DF in the study area, and recommend appropriate actions.

The identification of spatially relatively low- and high-risk clusters is integral to decision-making and prioritizing regions for immediate and/or gradual measures through policy implications [56,65,67,168], and as such, this study provides important references for this. The proposed framework integrates, *hot spot analysis* [169,170], *space–time cube analysis*, *emerging hot spots evaluation*, and *space–time prediction analytics*, see Figure 1. This integration is an integrated representation of the famous first law of geography presented by Waldo Tobler in 1970, which states “*everything is related to everything else*, *but near things are more related than distant things*” [171], and the second law of geography, also known as spatial heterogeneity (SLG), refers to the observation that “*conditions differ/change from place to place*” (non-stationarity) [172]. Similarly, the comprehensive mapping provided in this study is important to communicate the situation regarding DF, and can progressively be utilized in awareness through educational programs, which might result in better responses. The regions identified as the spatial–temporal hot spots should be prioritized by the concerned authorities for prevention and protection during future DF seasons. These higher concentration areas should further be evaluated for socio-economic characteristics of communities/neighborhoods as lower-income and education levels make people comparatively more susceptible to DF [144]. Hence, the study as such provides a baseline for these future studies to fill the knowledge gap in this field.

The conditions or potential factors associated with the spatial distribution of a vector disease vary from location to location, and hence, the site-specific identification of significant driving forces, such as the one presented in this study (Section 3.5, Table 3), behind the distribution of disease is particularly helpful for local-scale planning and decision-making [21,173,174,175]. In light of the fact that there is no specific medication available to treat DF infection, efforts to prevent it are one of the most effective measures [176,177]. In this context, while the factors associated with GWR modelling have significant implications for the study area, the I-SpaDE framework can be utilized in other cities of Pakistan and beyond. It is further notable that DF cases mainly occurred during periods with a mean temperature of 26.5 °C to 30 °C (Supplementary Figure S23), which is suitable for *Aedes aegypti* [178,179]. The most significant association between the spatial DF distribution and temperature, followed by vegetation cover and built-up area (Table 3), helps to design operative strategies to tackle DF at city scales. When there is such temperature suitability around vegetation and built-up neighborhoods, DF can be more prevalent and might trigger large epidemics in the absence of preventive measures. Additionally, there exists a possibility of applying the proposed framework to other vector diseases (e.g., malaria). However, this situation requires a reasonable amount of effort as well as the availability of data on disease and several associated factors.

## 5. Conclusions

Though the helpfulness of geospatial technology in disease mapping and health planning has recently become much more evident, the advancement in GIS and decision analytics tools necessitates the revisiting of typical in-practice disease assessment frameworks for robustness, reliability, and cost-effectiveness. Coupling different geospatial tools and space–time pattern mining techniques provides progressive opportunities in the context of vector disease prevention, control, and planning—with important public health implications. In this context, the study in hand proposed an *integrated spatial disease evaluation* (I-SpaDE) framework and demonstrated its application using Dengue Fever (DF) as an example vector disease in the second largest city of Pakistan, namely Lahore. The proposed framework advanced in-practice disease assessment approaches by integrating various spatial statistical models and space–time pattern analytics in a GIS environment, which could be used as a cost-effective public health planning tool. The application of the I-SpaDE framework showed that it can be very supportive for making policies and preventive measures within the built environment. DF showed significant spatial–temporal clustering during 2007–2008, 2010–2011, 2013, and 2016. The spatial proximities and heterogeneities in the DF cases and their hot spots were evident throughout the study area. On a temporal scale, the most affected months were September-November. The age groups of 11–20, 21–30, and 31–40 years were the main DF victims and within these groups, there was a substantial number of males. The DF remained prevalent in the *central functional area* of Lahore until 2011, but the incidence decreased in those areas from 2013 and emerged in the outskirts of the major urban areas, which remained there until 2016. It is further noted that space–time prediction zones are also nearby the *central functional area* and could be the next DF-affected places.

The indication of the temperature as the most significantly associated factor with the spatial distribution of DF provides insights to take appropriate measures in regions with suitable temperatures. While the spatial assessment made in the study can lead to questions along the lines of “where to put the preventive efforts”, the space–time analysis further makes it convenient to answer “when” during DF season. This shows the practical implications of the results and the framework for smart decision-making, effective resource allocation, and policy development within an urban area. Similarly, the proposed I-SpaDE framework is extensible, replicable, and adaptable in other tropical and sub-tropical regions of the world where vector diseases such as DF are common. However, this might require a reasonable effort and proper data understanding.

## Figures and Tables

**Table 1:** Justification of vital indicators used in the GWR model.

Dengue Supportive Factor	Factor Computation	Explanation of Significance	Justification/Reference
Built-up area	*The Normalized Difference Built-up index (NDBI)*	It is widely indicated that more urban areal development or built-up land positively supports the *Aedes aegypti* (Urban mosquito) presence. This mosquito thrives in urban settings where there is infrastructural proximity. The indoor places are among the other resting places of *Aedes aegypti*; the host is at constant risk of frequent bites and infection inside such indoor spaces.	Wu et al. [125]; Acharya et al. [126]; Estallo et al. [127]; Kalbus et al. [128]; Nakhapakorn and Tripathi [129]; Schoof [130]; Dzul-Manzanilla et al. [131]
Population	The data downloaded from *European Commission’s Global Human Settlement* data portal; Schiavina et al. [132]	Dengue is an urban disease; due to the high density of human populations and increased adaptation of *Aedes aegypti* to densely populated environments. Population density is an important indicator in dengue assessments because the moving of the population from place to place plays a crucial role in dengue epidemics.	Marti et al. [133]; Kalbus et al. [128]; Acharya et al. [126]; Estallo et al. [127]; Lin and Wen [134]; Wu et al. [125]
Vegetation	*The Normalized Difference Vegetation Index (NDVI)*	The *Aedes aegypti* mosquitoes remain active during diurnal times and their resting habitats are typically associated with vegetation (during daytimes)—that provides ideal shade and, therefore, a microclimate— which is cooler than those in open lands, e.g., bare soil and built-up areas.	Imran et al. [21]; Estallo et al. [127]; Acharya et al. [126]; Tariq and Zaidi [135]
Land Surface Temperature (LST)	Calculated from Landsat 5 (*Thematic Mapper (TM)*); *Thermal Band (10.40–12.50 µm)* Nakhapakorn et al. [136]	Temperature is considered the paramount meteorological factor influencing ecological distributions of *Aedes aegypti* mosquitoes. Land surface temperature is used by numerous researchers to assess dengue-related associations.	Tsai et al. [137]; Tariq and Zaidi [135]; Acharya et al. [126]; Imran et al. [21]
Water	Computed through the *Normalized Difference Water Index (NDWI)*	Dengue is one of the water-associated diseases and water proximity could be an important factor in such heterogeneity-based assessments. Water plays a vital role in dengue mosquitoes’ breeding, especially when combined with other factors such as suitable temperature and vegetation.	Dickin et al. [138]; Estallo et al. [127]; Tariq and Zaidi [135]; Li et al. [139]; Acharya et al. [126]
Moisture	Computed through the *Normalized Difference Moisture Index (NDMI)*	The mosquitoes’ vector breeding at any location highly depends on moisture, water, temperature, and vegetation. High moisture levels with high-temperature conditions are climatically optimal for the distribution of *Aedes aegypti*, which is connected to Dengue Fever.	Kumar and Agrawal [140]; Sintayehu et al. [141]

**Table 2:** Mann–Kendall trend test showing dengue’s space–time trend from 2007 to 2016.

Year	Incidents	Trend	Trend Statistic	*p*-Value	Interpretation
2007	241	Increasing	2.7563	0.0058	Reject *H*_0_
2008	1180	Increasing	3.5523	0.0004	Reject *H*_0_
2009	89	Not Significant	0.2772	0.7816	Accept *H*_0_
2010	3580	Increasing	5.1586	0.0000	Reject *H*_0_
2011	11,283	Increasing	3.3984	0.0007	Reject *H*_0_
2012	124	Increasing	2.1497	0.0316	Reject *H*_0_
2013	1512	Increasing	5.5800	0.0000	Reject *H*_0_
2014	83	Increasing	4.2038	0.0000	Reject *H*_0_
2015	146	Increasing	2.8246	0.0047	Reject *H*_0_
2016	1111	Increasing	2.5990	0.0093	Reject *H*_0_

**Table 3:** Results from the GWR model for grid-based and administrative unit-based analysis.

Variable	Grid-Based				Administrative Unit-Based (UCs)			
	Intercept	NDBI	NDVI	LST	Intercept	NDBI	NDVI	LST
Mean of βs	6.473	0.181	0.272	27.790	15.248	0.167	0.213	20.529
SD of βs	8.059	0.038	0.118	0.922	22.333	0.179	0.169	23.268
Minimum	0.001	−0.116	−0.067	23.683	0.000	0.002	0.004	0.208
Maximum	70.009	0.402	0.574	31.646	145.439	1.454	0.839	195.225
Median	3.137	0.187	0.282	27.935	8.630	0.133	0.194	17.042
SE	0.093	0.000	0.001	0.011	1.817	0.015	0.014	1.894
Adjusted R^2^	0.84				0.73			
Akaike information criterion (AIC)	39,445.90				1175.44			

Note: SD represents standard deviation and SE represents standard error.

## Data Availability

The geocoded datasets analyzed during the current research are available from the corresponding author on reasonable request.

## Supplementary Materials

The following are available online at https://www.mdpi.com/article/10.3390/ijerph182212018/s1, Figure S1: Incremental Spatial Autocorrelation results for 2007; Figure S2: Incremental Spatial Autocorrelation results for 2008; Figure S3: Incremental Spatial Autocorrelation results for 2009; Figure S4: Incremental Spatial Autocorrelation results for 2010; Figure S5: Incremental Spatial Autocorrelation results for 2011; Figure S6: Incremental Spatial Autocorrelation results for 2012; Figure S7: Incremental Spatial Autocorrelation results for 2013; Figure S8: Incremental Spatial Autocorrelation results for 2014; Figure S9: Incremental Spatial Autocorrelation results for 2015; Figure S10: Incremental Spatial Autocorrelation results for 2016; Figure S11: Incremental Spatial Autocorrelation results for January; Figure S12: Incremental Spatial Autocorrelation results for February; Figure S13: Incremental Spatial Autocorrelation results for March; Figure S14: Incremental Spatial Autocorrelation results for April; Figure S15: Incremental Spatial Autocorrelation results for May; Figure S16: Incremental Spatial Autocorrelation results for June; Figure S17: Incremental Spatial Autocorrelation results for July; Figure S18: Incremental Spatial Autocorrelation results for August; Figure S19: Incremental Spatial Autocorrelation results for September; Figure S20: Incremental Spatial Autocorrelation results for October; Figure S21: Incremental Spatial Autocorrelation results for November; Figure S22: Incremental Spatial Autocorrelation results for December; Figure S23: Dengue cases in autumn season (2007–2016) vs. corresponding LST (2007–2016); Table S1: Descriptive statistics of statistically significant hotspots and cold-spots on annual basis; Table S2: Descriptive statistics of statistically significant hotspots and cold-spots on monthly basis.
