# Supplementary material for: Integrating Spatial Modelling and Space–Time Pattern Mining Analytics for Vector Disease-Related Health Perspectives: A Case of Dengue Fever in Pakistan
Source: Int J Environ Res Public Health. 2021 Nov 16;18(22):12018. doi: 10.3390/ijerph182212018 (PMC8618682; doi:10.3390/ijerph182212018)
Supplement: Supplementary file 1 [file ijerph-18-12018-s001.zip › ijerph-1368038-supplementary.pdf]

Supplementary Information For

Integrating Spatial Modelling and Space–time Pattern Mining Analytics for Vector Disease-Related Health Perspectives: A Case of Dengue Fever in Pakistan

Syed Ali Asad Naqvi [1,*,†], Muhammad Sajjad [2,†], Liaqat Ali Waseem [1], Shoaib Khalid [1], Saima Shaikh [3] and Syed Jamil Hasan Kazmi [3]

[1] Department of Geography, Government College University Faisalabad, Faisalabad 38000, Pakistan; drliaqataliwaseem@gcuf.edu.pk (L.A.W.); shoaibkhalid@gcuf.edu.pk (S.K.)
[2] Department of Geography, Hong Kong Baptist University, Hong Kong; mah.sajjad@hotmail.com
[3] Department of Geography, University of Karachi, Karachi 75270, Pakistan; saima-ku@uok.edu.pk (S.S.); jkazmi@usa.net (S.J.H.K.)
* Correspondence: draliasad@gcuf.edu.pk; Tel.: +92-306-6741774
† These authors contributed equally to this study.

Supplementary Figures

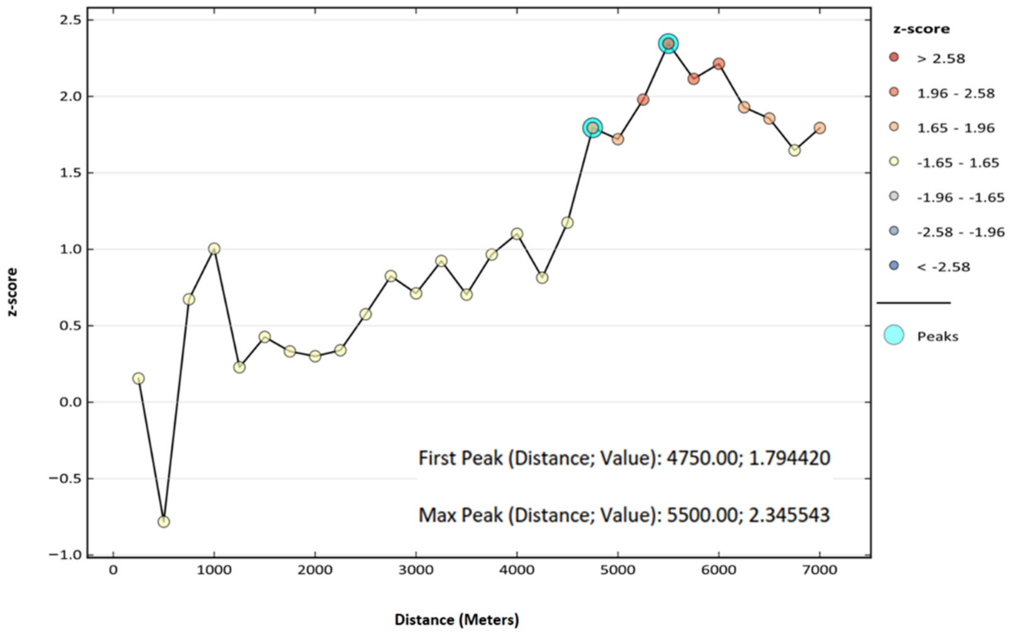

Supplementary Figure S1. Incremental Spatial Autocorrelation results for 2007.

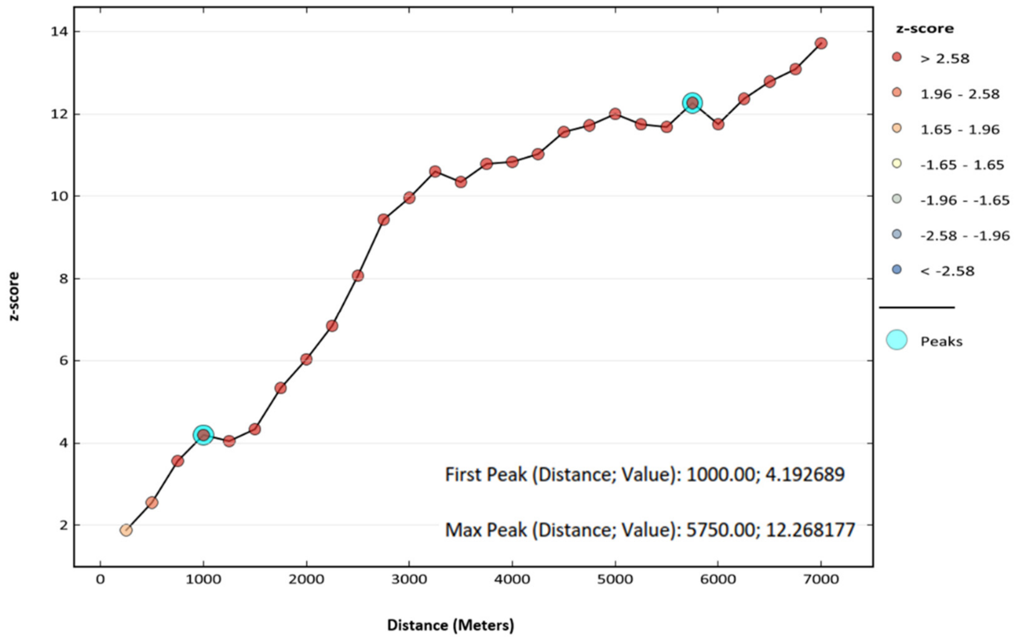

Supplementary Figure S2. Incremental Spatial Autocorrelation results for 2008.

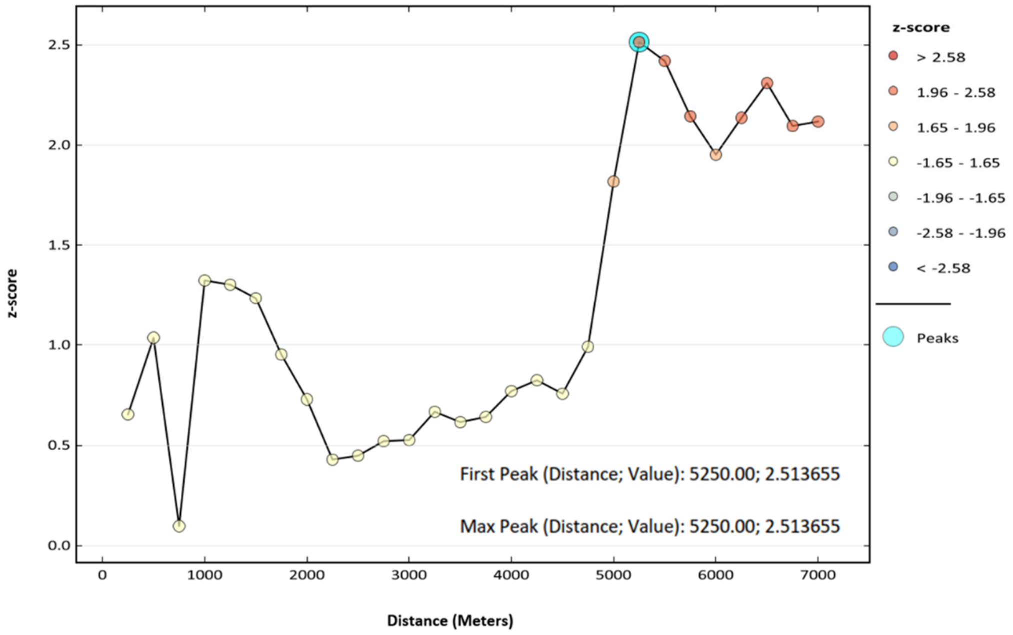

Supplementary Figure S3. Incremental Spatial Autocorrelation results for 2009.

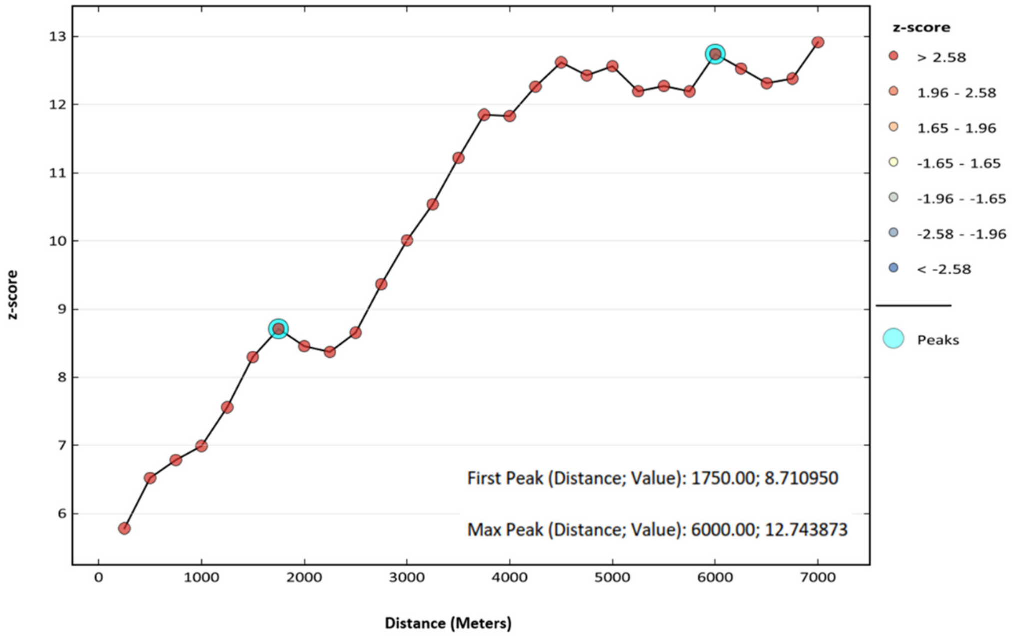

Supplementary Figure S4. Incremental Spatial Autocorrelation results for 2010.

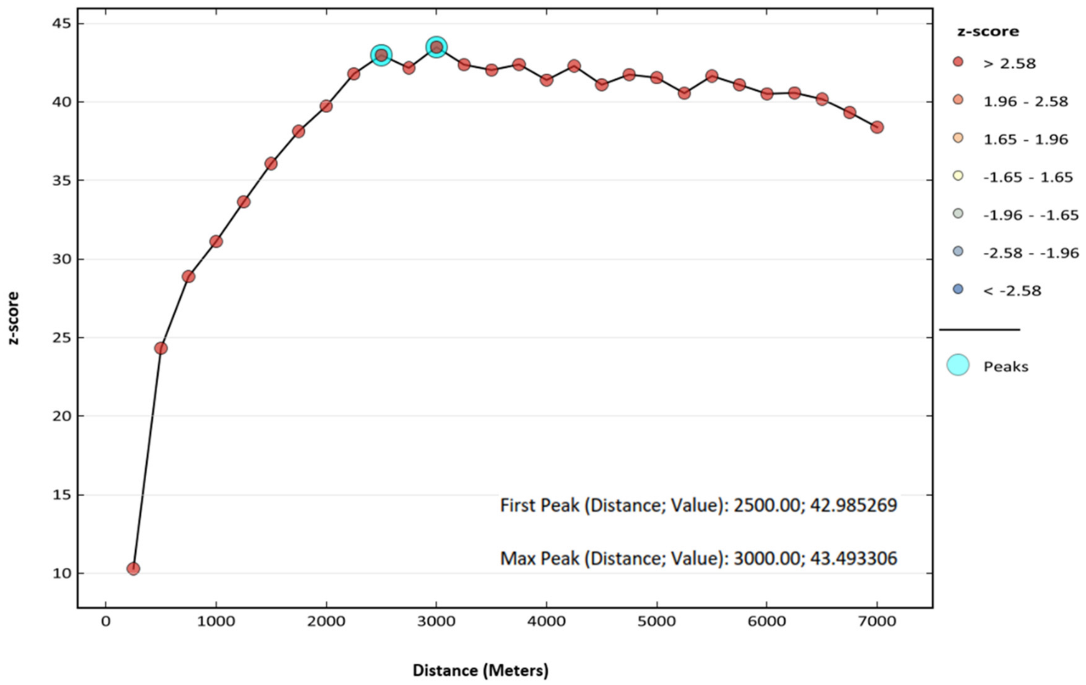

Supplementary Figure S5. Incremental Spatial Autocorrelation results for 2011.

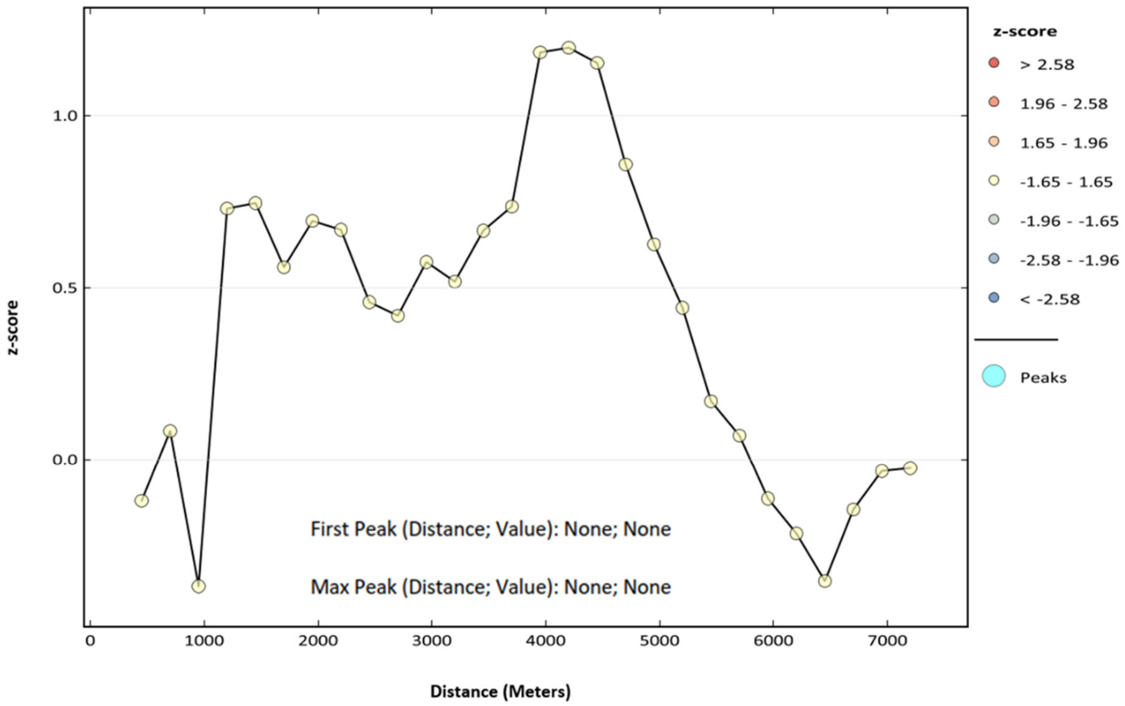

Supplementary Figure S6. Incremental Spatial Autocorrelation results for 2012.

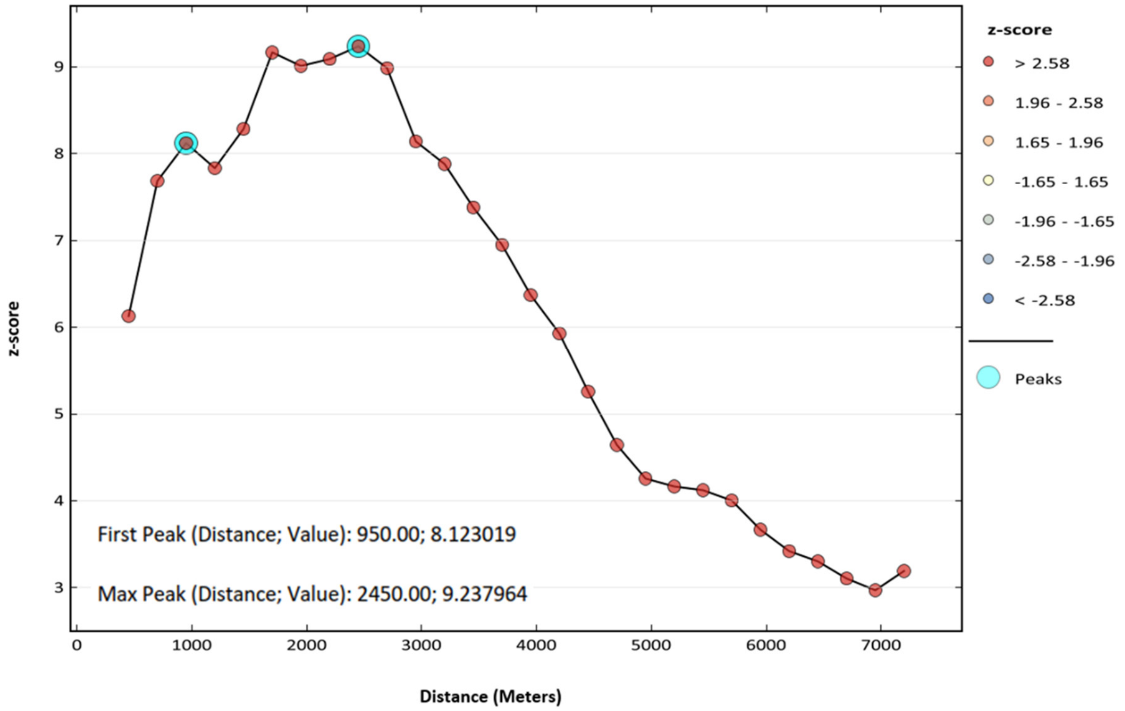

Supplementary Figure S7. Incremental Spatial Autocorrelation results for 2013.

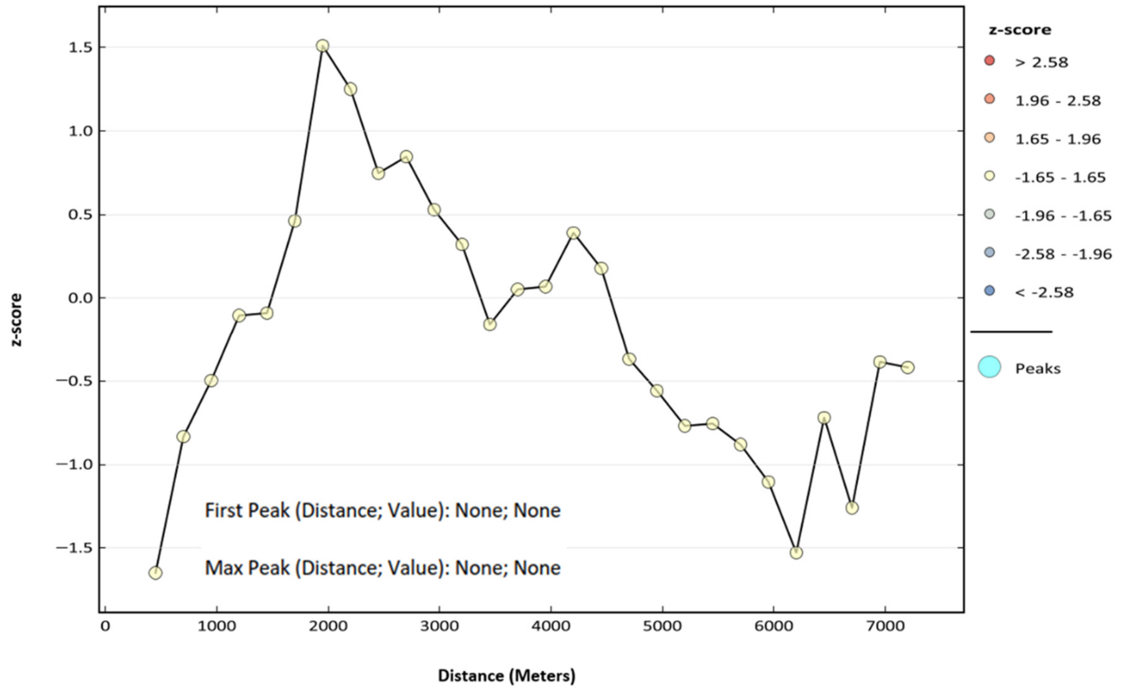

Supplementary Figure S8. Incremental Spatial Autocorrelation results for 2014.

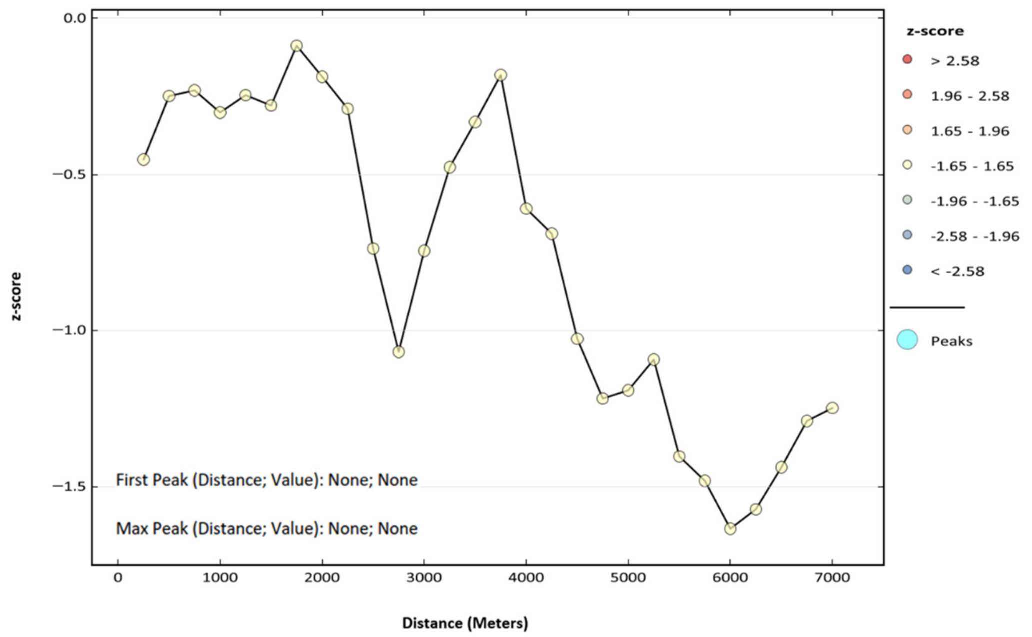

Supplementary Figure S9. Incremental Spatial Autocorrelation results for 2015.

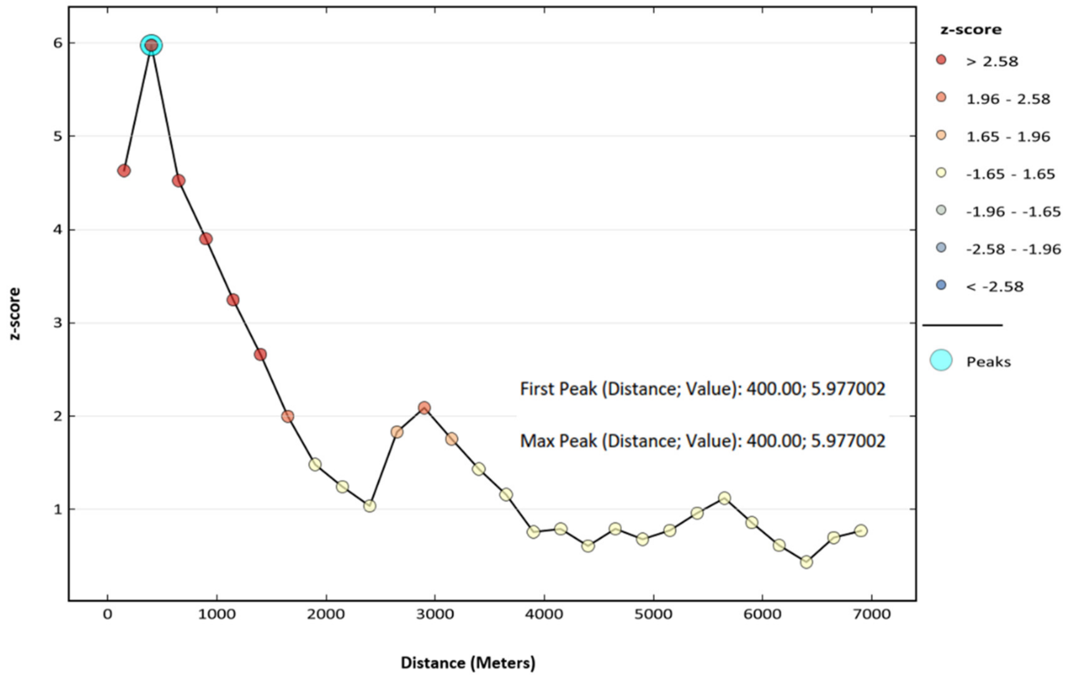

Supplementary Figure S10. Incremental Spatial Autocorrelation results for 2016.

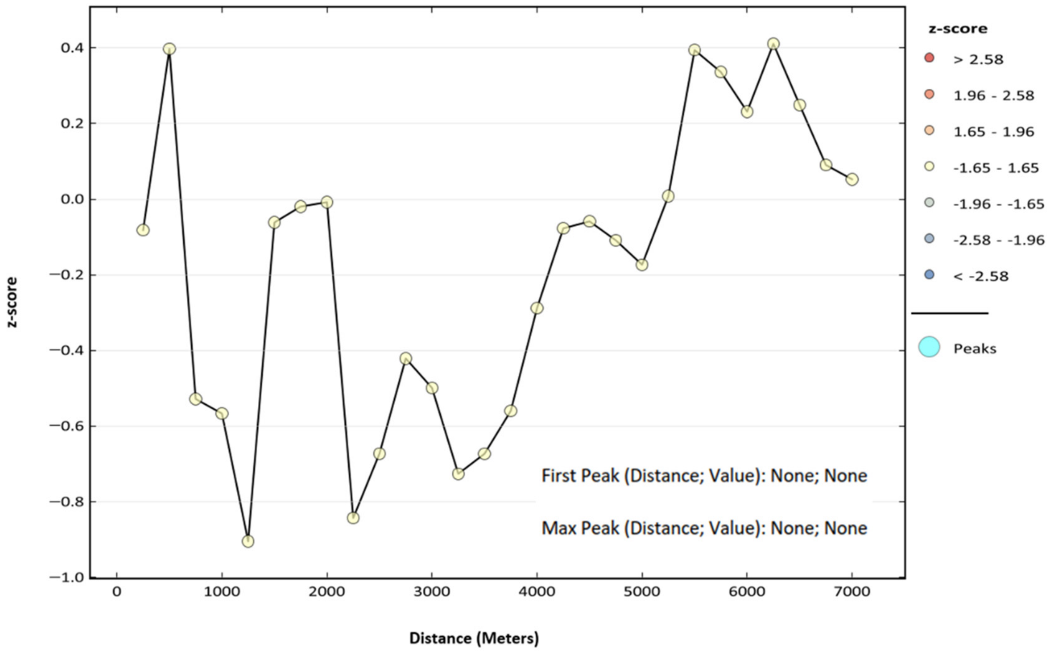

Supplementary Figure S11. Incremental Spatial Autocorrelation results for January.

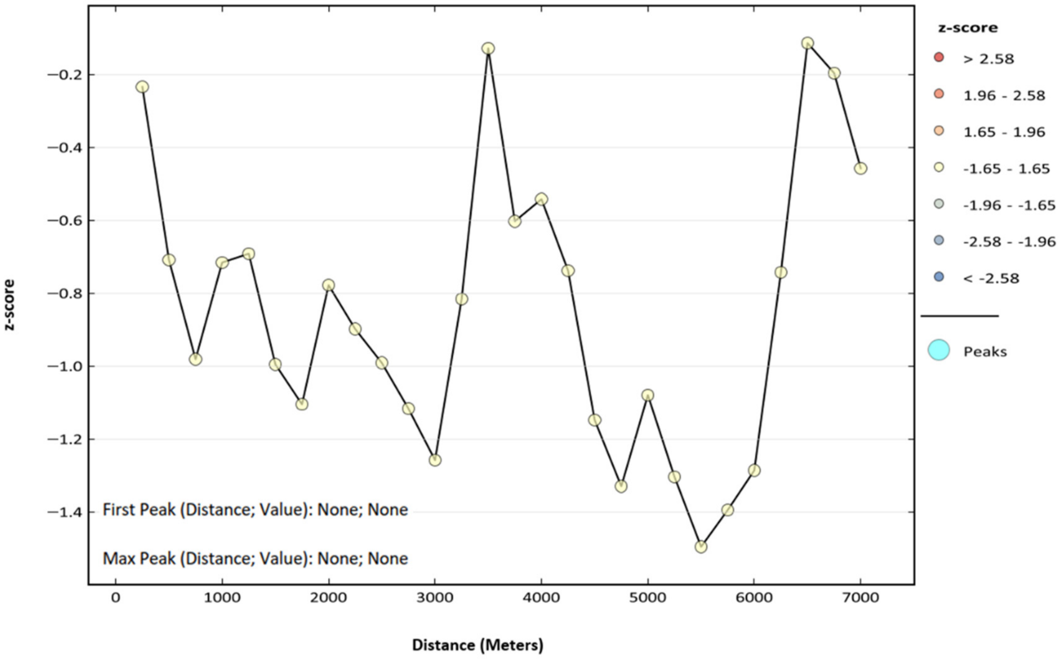

Supplementary Figure S12. Incremental Spatial Autocorrelation results for February.

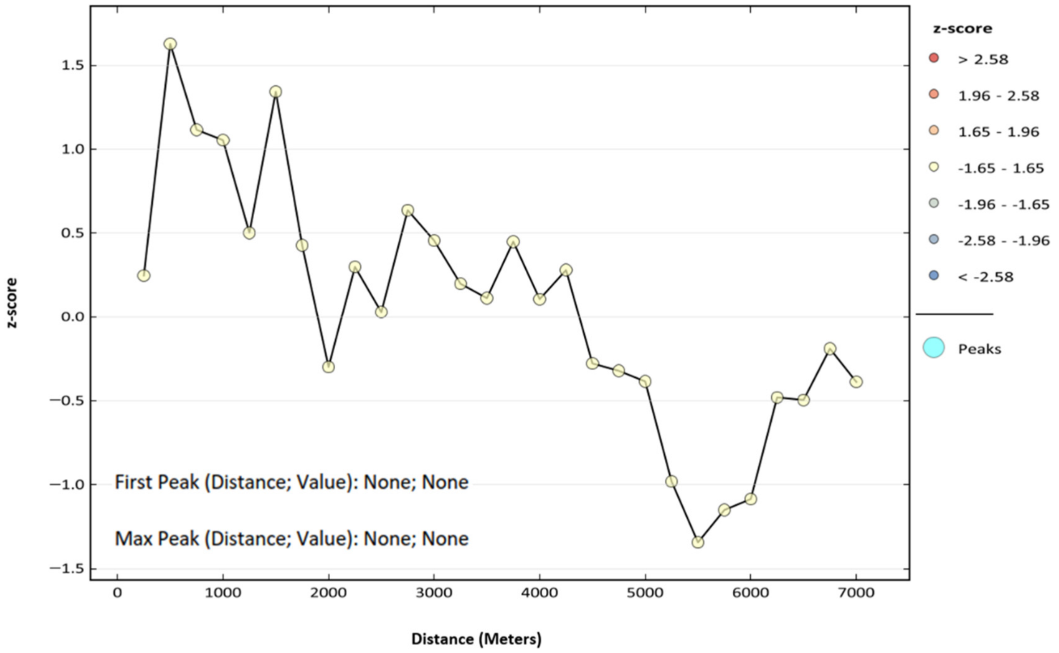

Supplementary Figure S13. Incremental Spatial Autocorrelation results for March.

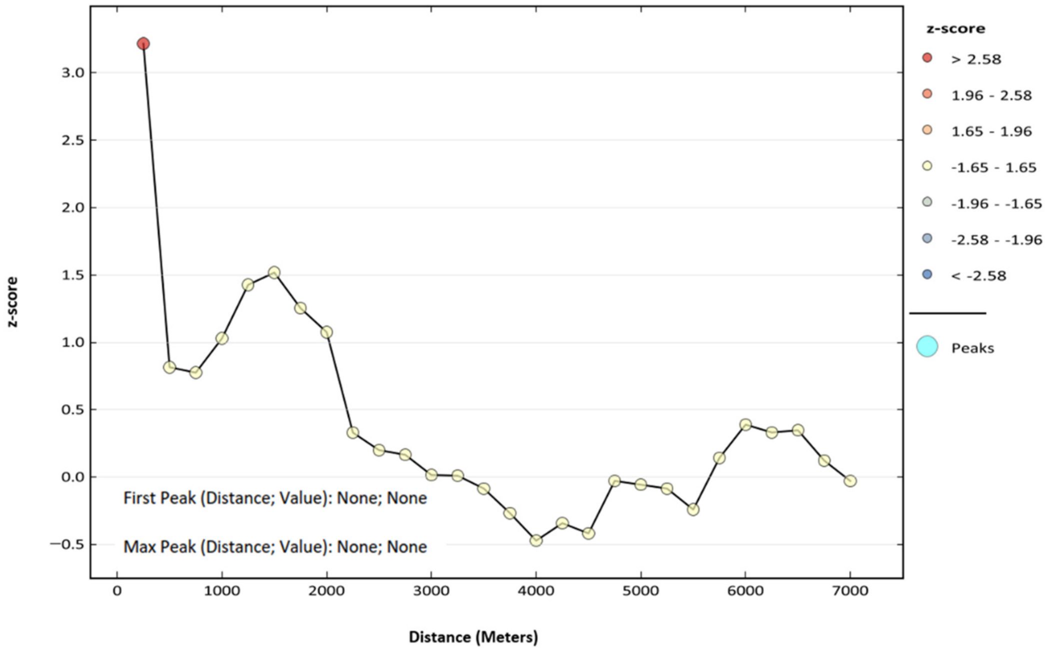

Supplementary Figure S14. Incremental Spatial Autocorrelation results for April.

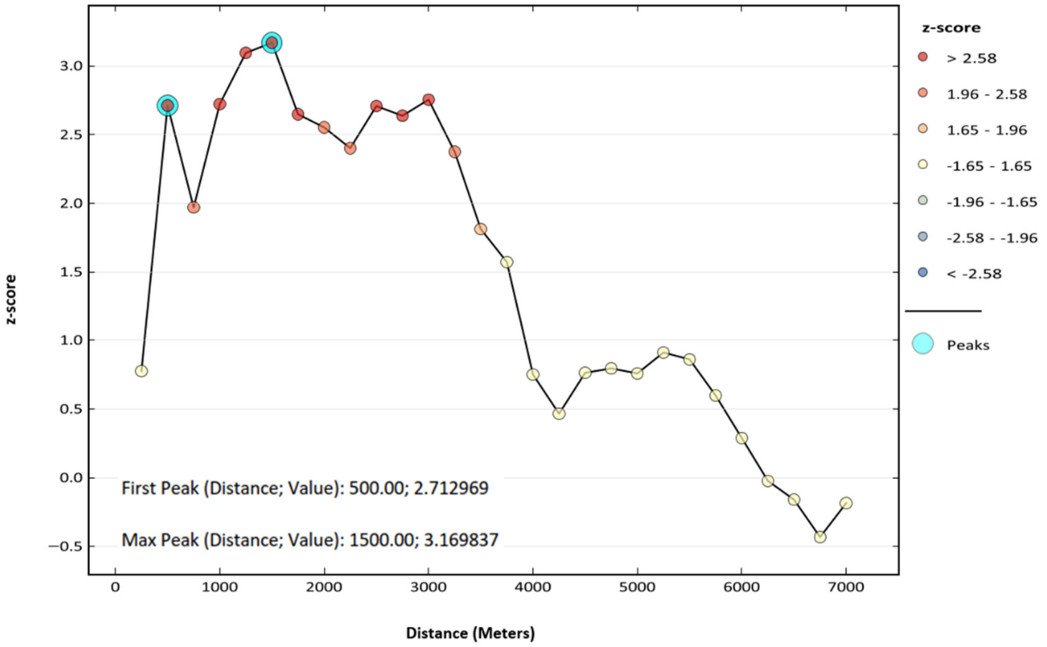

Supplementary Figure S15. Incremental Spatial Autocorrelation results for May.

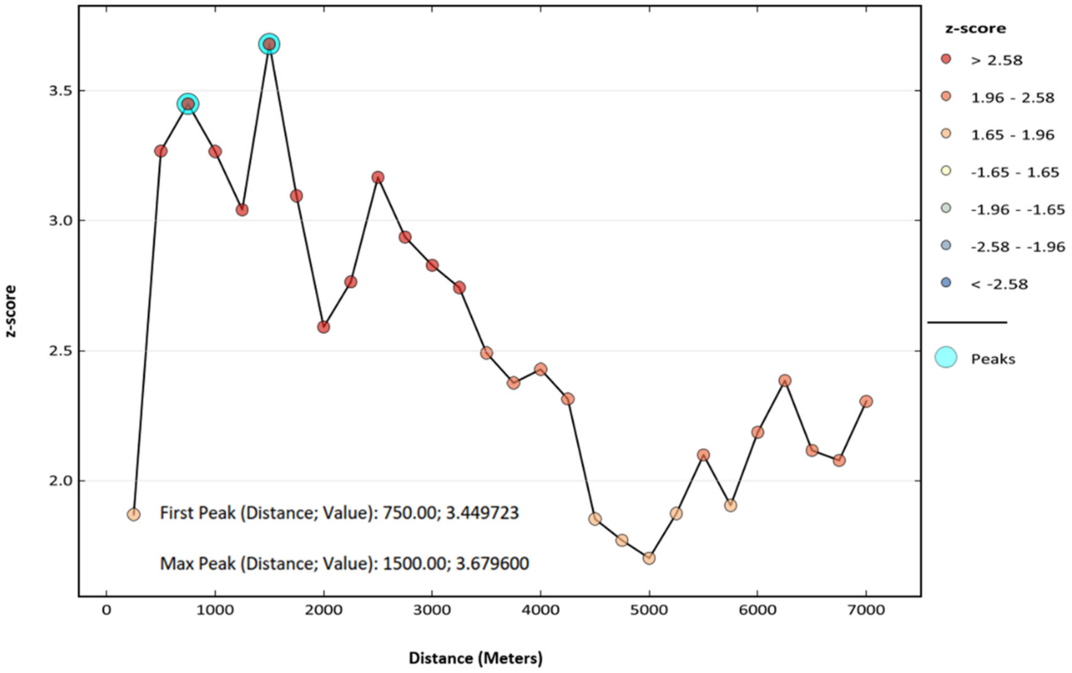

Supplementary Figure S16. Incremental Spatial Autocorrelation results for June.

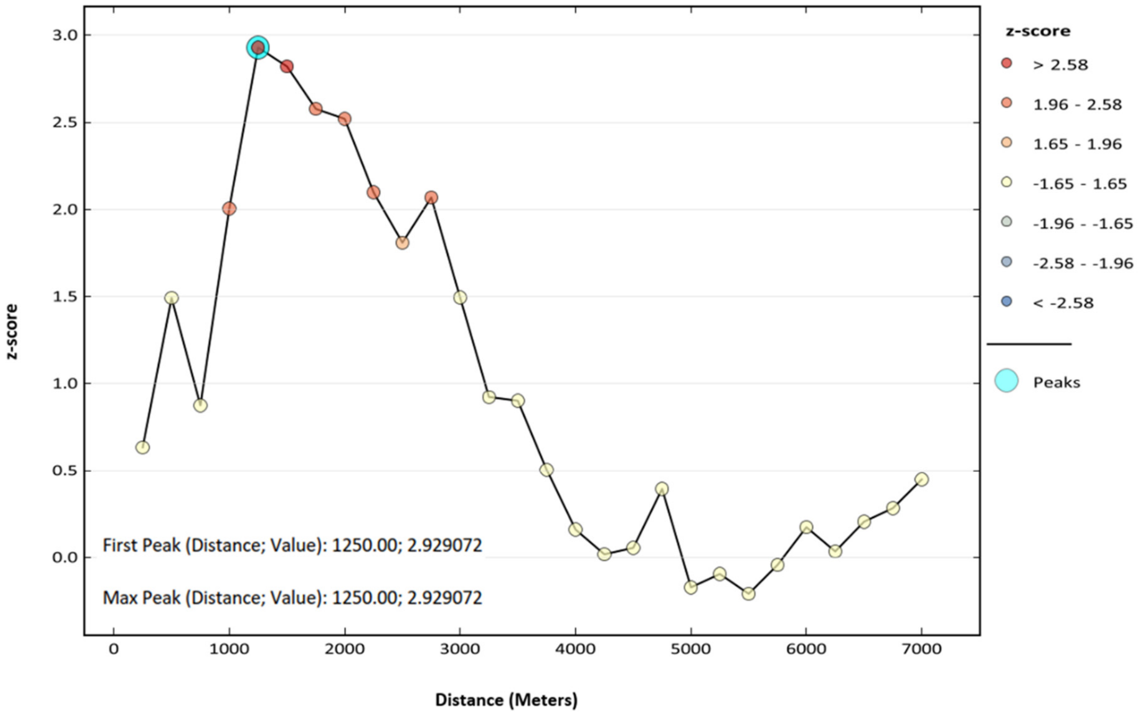

Supplementary Figure S17. Incremental Spatial Autocorrelation results for July.

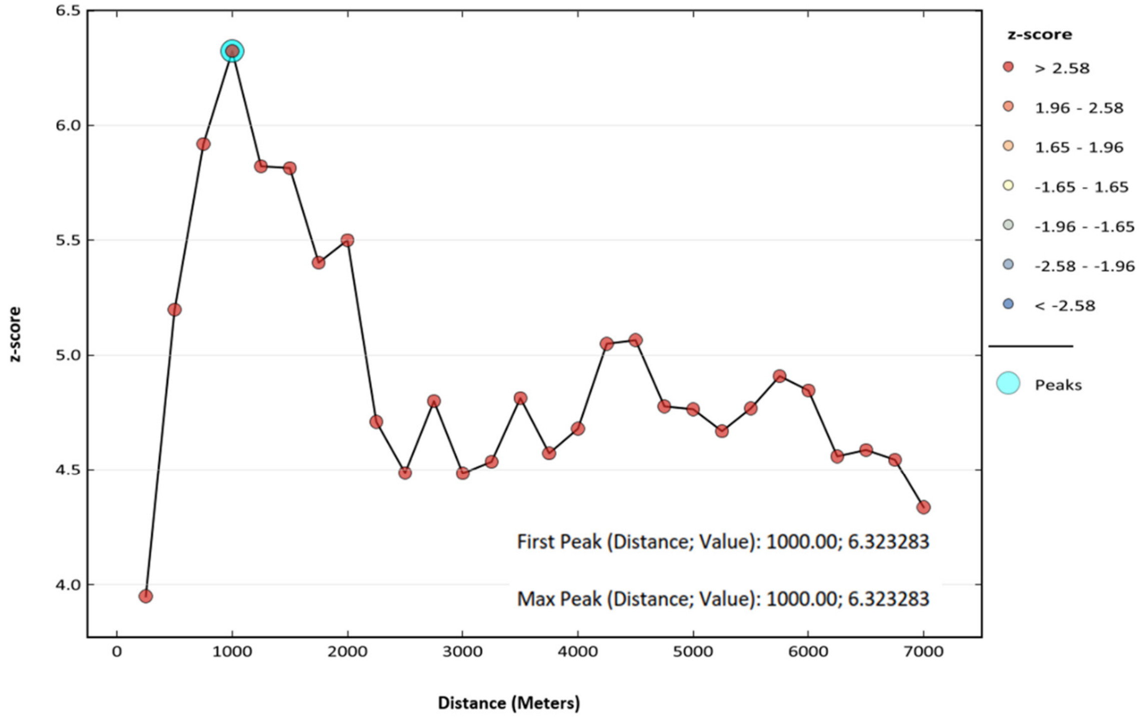

Supplementary Figure S18. Incremental Spatial Autocorrelation results for August.

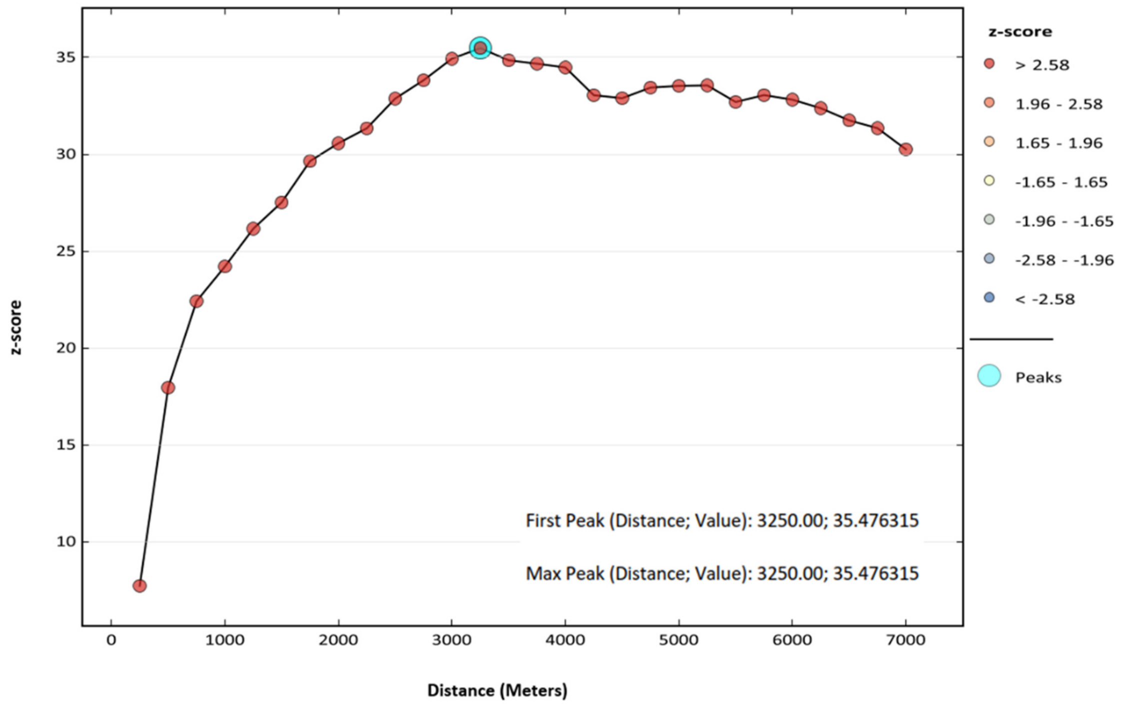

Supplementary Figure S19. Incremental Spatial Autocorrelation results for September.

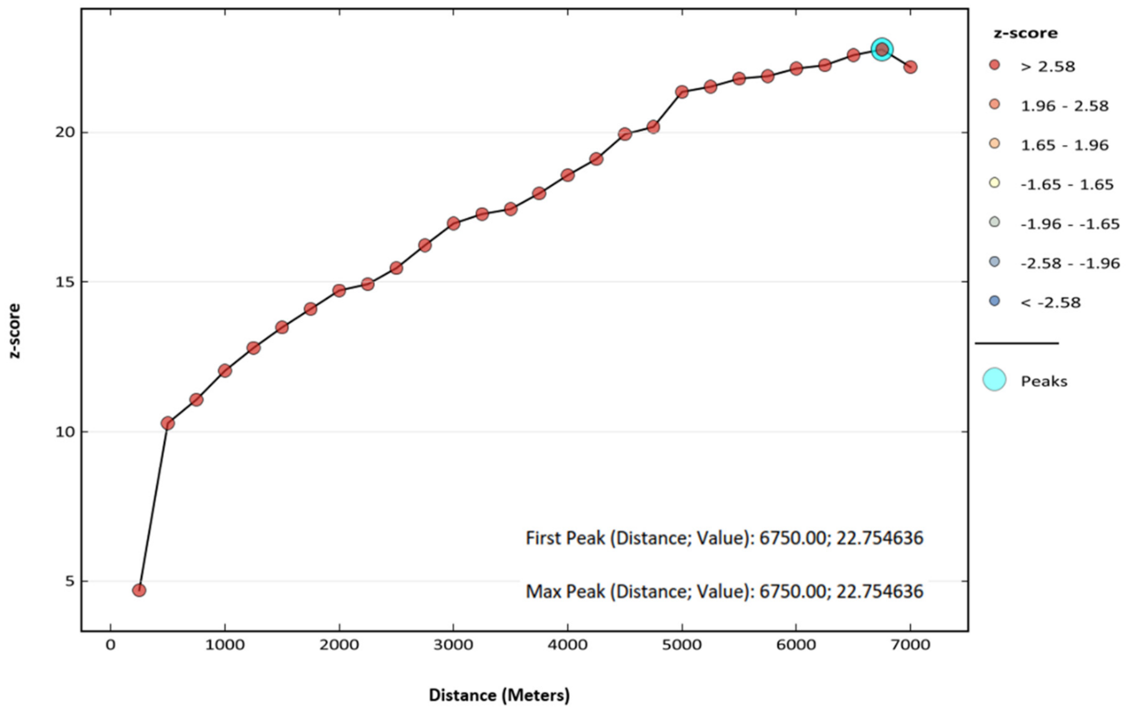

Supplementary Figure S20. Incremental Spatial Autocorrelation results for October.

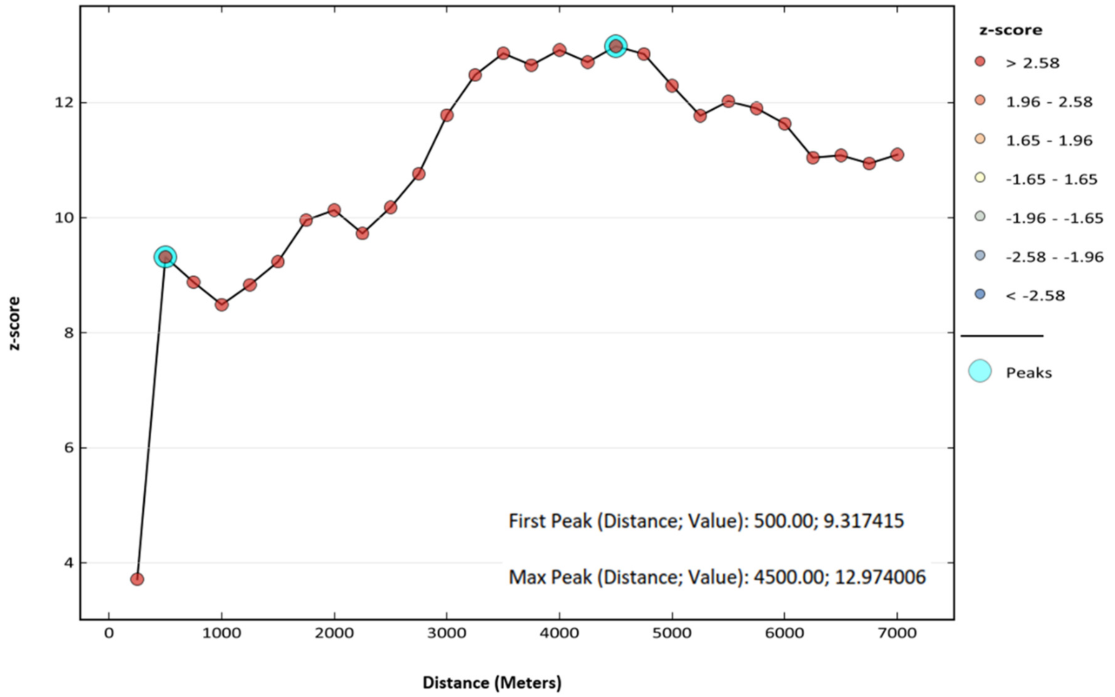

Supplementary Figure S21. Incremental Spatial Autocorrelation results for November.

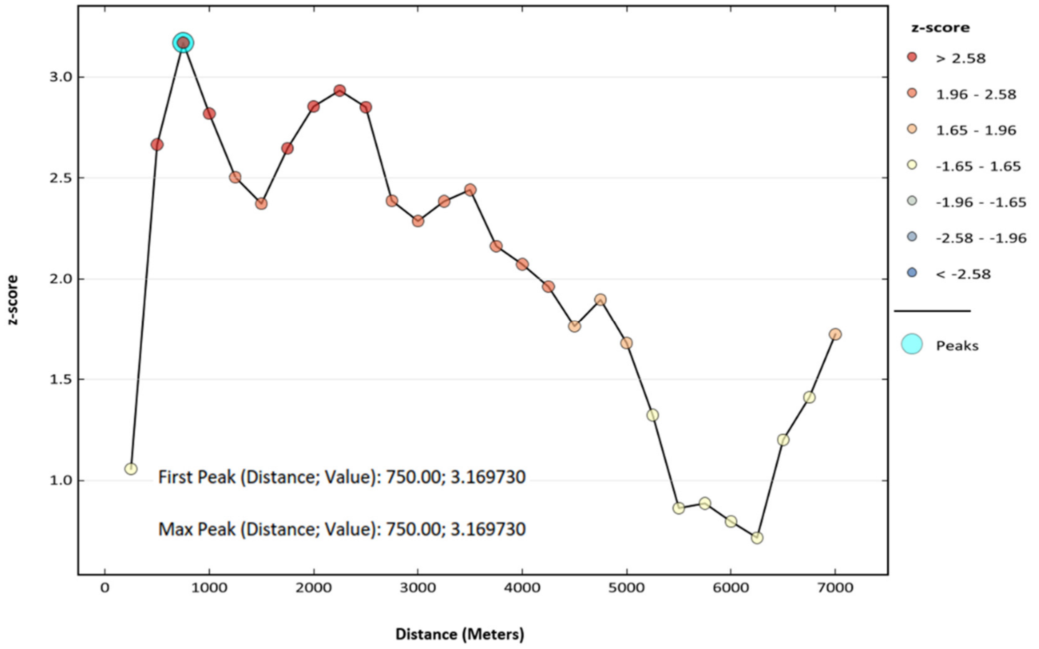

Supplementary Figure S22. Incremental Spatial Autocorrelation results for December.

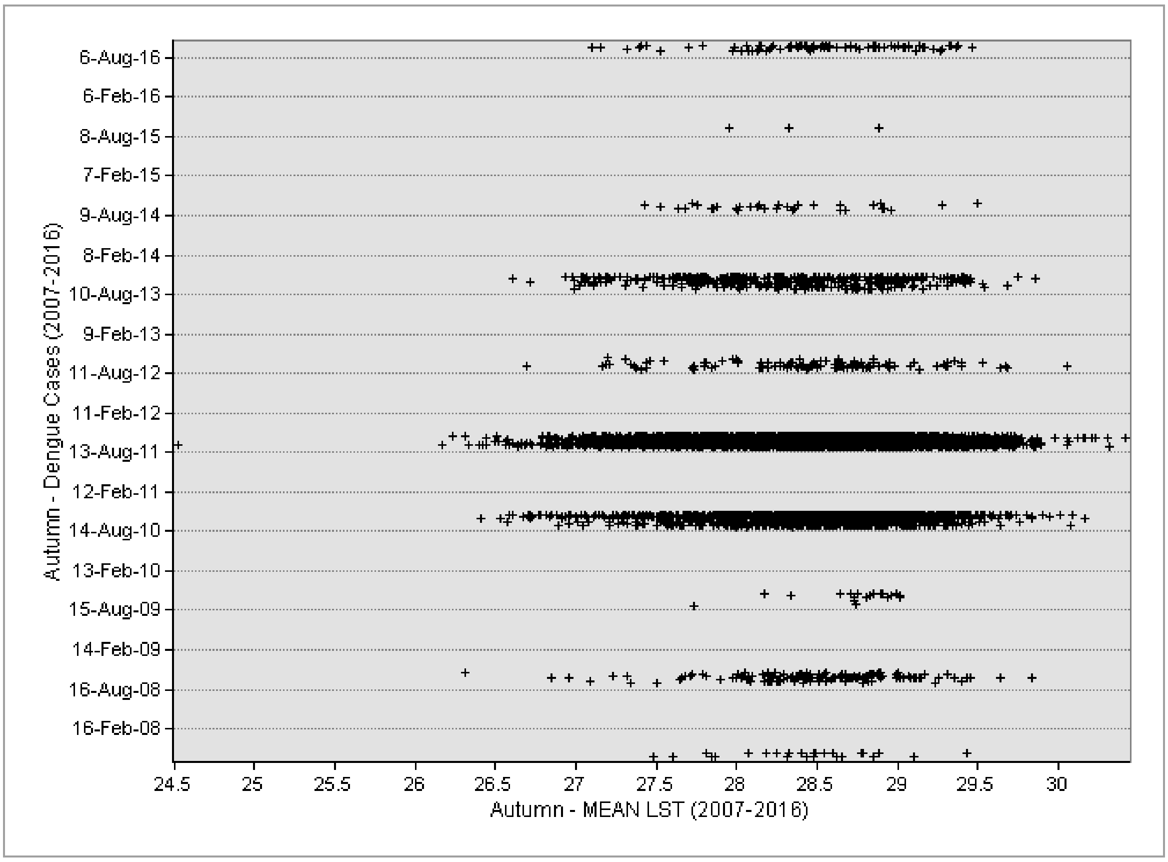

Supplementary Figure S23. Dengue cases in autumn season (2007-2016) vs corresponding LST. (2007-2016).

Supplementary Tables

Supplementary Table S1. Descriptive statistics of statistically significant hotspots and cold-spots on annual basis.

Period	Hotspots (*z-score*)				Cold-spots (*z-score*)			
	Significant *z-score* (Min-Max)	Mean	SD	Total Bins	Significant *z-score* (Min-Max)	Mean	SD	Total Bins
2007	2.19 to 3.34	2.57	0.29	54	---	---	---	None
2008	1.72 to 7.79	5.10	1.56	476	-6.41 to -1.71	-4.37	1.23	112
2010	1.78 to 9.35	5.99	1.79	816	-5.82 to -1.77	-4.08	1.24	349
2011	1.83 to 18.40	10.19	5.26	1301	-8.30 to -1.84	-4.03	1.51	1064
2013	1.97 to 7.30	3.69	0.87	309	-3.43 to -1.98	-2.56	0.32	145
2016	2.51 to 6.28	3.87	1.12	58	None	None	None	None

Supplementary Table S2. Descriptive statistics of statistically significant hotspots and cold-spots on monthly basis.

Period	Hotspots (*z-score*)				Cold-spots (*z-score*)			
	Significant *z-score* (Min-Max)	Mean	SD	Total Bins	Significant *z-score* (Min-Max)	Mean	SD	Total Bins
August	2.31 to 4.95	3.30	0.65	219	-2.37 to -2.36	-2.365	0.005	02
September	1.89 to 18.22	10.02	4.79	709	-5.62 to -1.87	-3.10	0.95	841
October	1.69 to 13.64	8.11	3.12	1129	-8.83 to -1.71	-5.46	1.81	806
November	1.82 to 10.94	5.90	2.37	735	-5.53 to -1.82	-3.20	0.99	586
December	2.13 to 3.87	2.93	0.43	211	-2.81 to -2.15	-2.41	0.20	11
